# Supplementary material for: The glycan alphabet is not universal: a hypothesis
Source: Microb Genom. 2020 Oct 13;6(11):mgen000452. doi: 10.1099/mgen.0.000452 (PMC7725333; doi:10.1099/mgen.0.000452)
Supplement: Supplementary material 1 [file mgen-6-452-s001.pdf]

SUPPLEMENTARY MATERIAL

The glycan alphabet is not universal: a hypothesis

Jaya Srivastava[1]*, P. Sunthar[2] and Petety V. Balaji[1]

[1]Department of Biosciences and Bioengineering, Indian Institute of Technology Bombay, Powai, Mumbai 400076, India

[2]Department of Chemical Engineering, Indian Institute of Technology Bombay, Powai, Mumbai 400076, India

*Corresponding author
Email: jaya_srivastava@iitb.ac.in

CONTENTS

Data	Description
Figure S1	Number of organisms with different number of strains sequenced
Figure S2	Biosynthesis pathways
Figure S3	Bit score distribution plots for hits of various pairs of profiles
Figure S4	Proteome sizes for different number of monosaccharides
Figure S5	Prevalence of monosaccharides in species versus that in genomes
Table S1	Tools and databases used in this study
References	References cited in Table S1
Table S2	Comparison of the precursor and nucleotide used for the biosynthesis of two enantiomers of a monosaccharide
Flowchart S1	Procedure used to generate HMM profiles
Flowchart S2	Precedence rules for assigning annotation to proteins that are hits to two or more profiles and/or BLASTp queries
References	References to the research articles which describe the pathways (or enzymes of the pathways) of monosaccharide biosynthesis. These formed the basis for generating HMM profiles and choosing BLASTp queries.

MS-EXCEL file provided separately: Supplementary Data.xlsx

Worksheet1	Details of HMM profiles
Worksheet2	Details of BLASTp queries
Worksheet3	Prevalence of monosaccharides in genomes / species
Worksheet4	Abbreviated names of monosaccharides
Worksheet5	Enzyme types, enzymes and monosaccharide groups
Worksheet6	Precursors of various monosaccharides

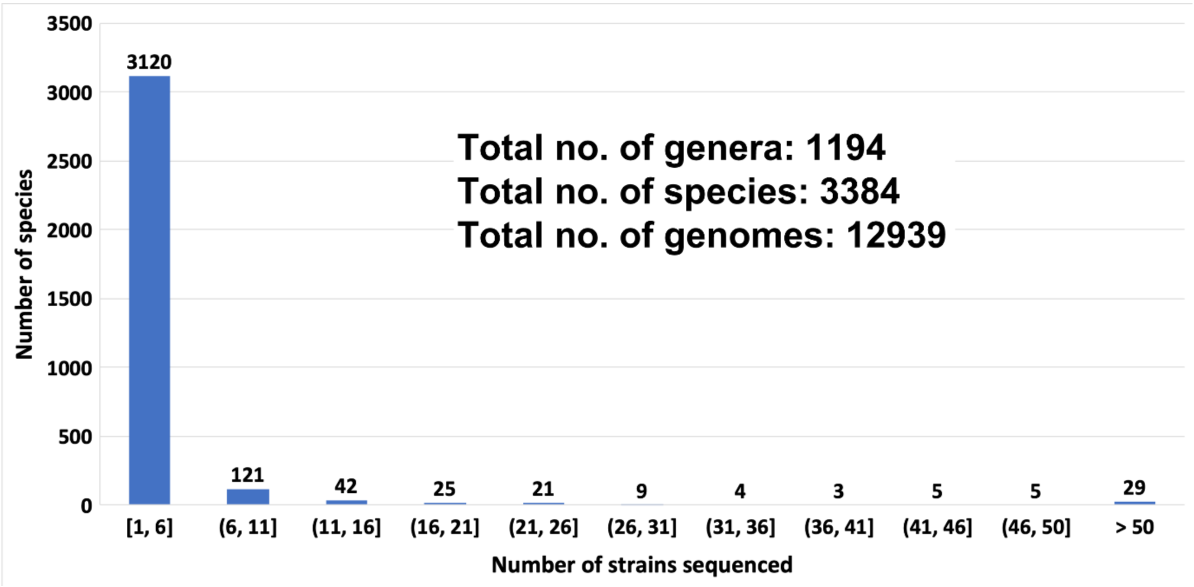

Figure S1 The number of species for which different number of strains are sequenced. Six or fewer strains are sequenced for most of the species. On the other hand, more than 50 strains are sequenced for 29 species. *Escherichia coli* and *Salmonella enterica* have the highest number of sequenced strains (714 and 602, respectively). Genus and species names are not known for 45 endosymbionts; only their host name is known e.g., Legionella endosymbiont. Each such case is considered as a distinct species.

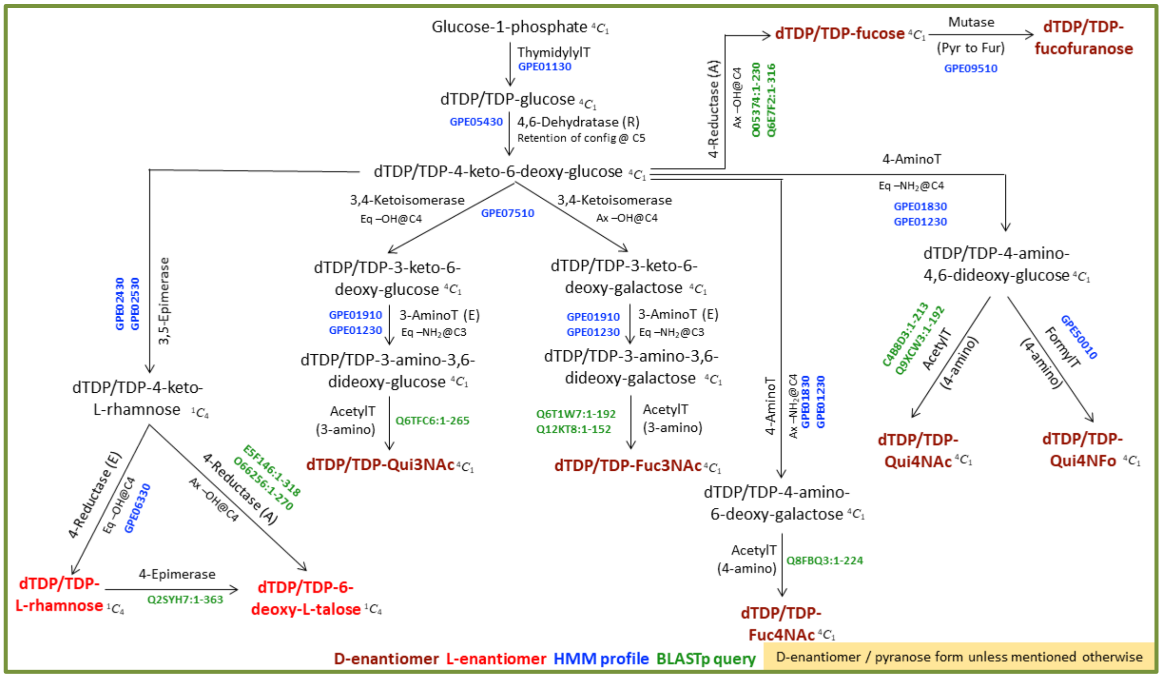

Figure S2a TDP-/dTDP-linked monosaccharides derived from glucose-1-phosphate. Abbreviated names are used for some of the monosaccharides. Full names of these are given in Supplementary_data.xlsx:Worksheet4.

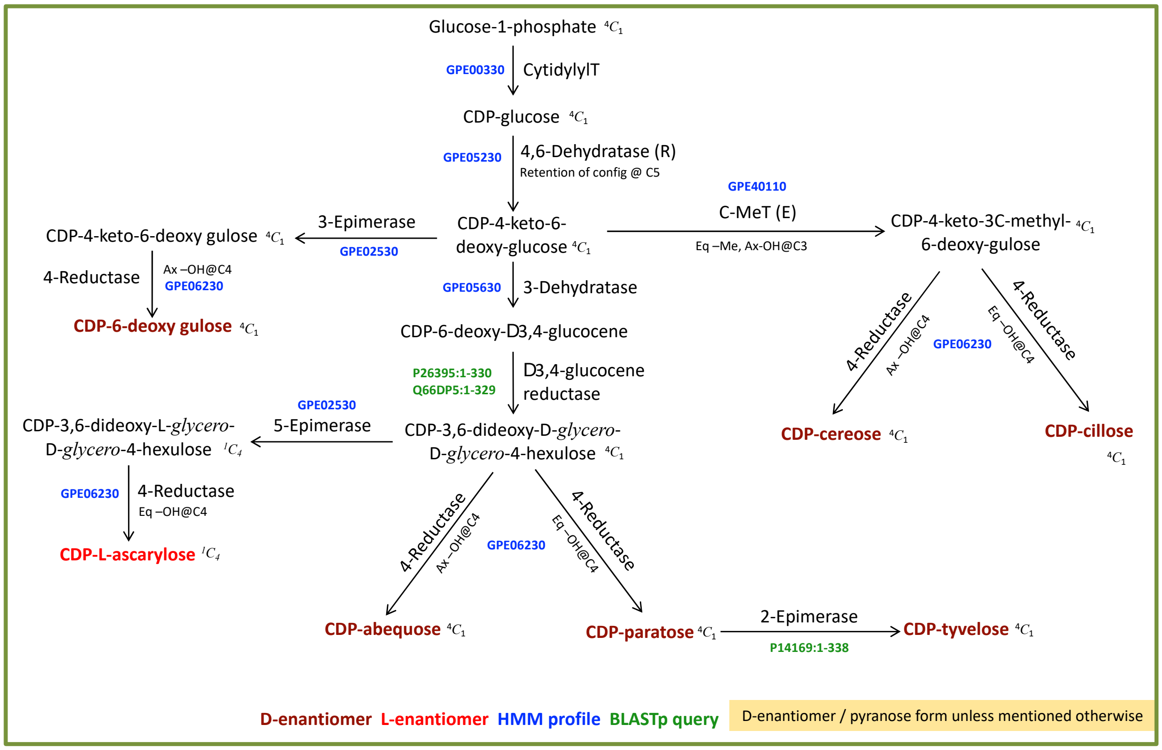

Figure S2b CDP-linked monosaccharides derived from glucose-1-phosphate. Abbreviated names are used for some of the monosaccharides. Full names of these are given in Supplementary_data.xlsx:Worksheet4.

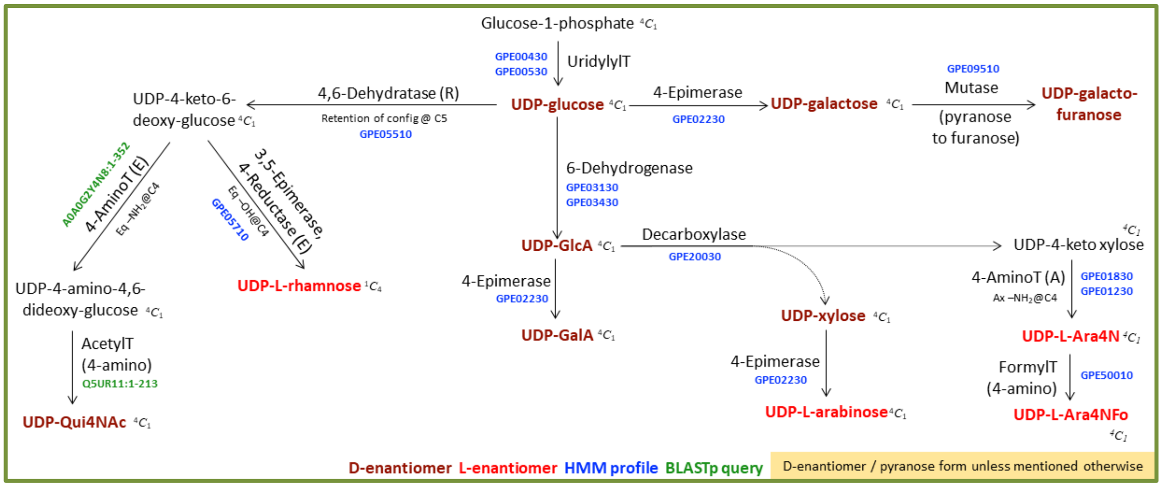

Figure S2c UDP-linked monosaccharides derived from glucose-1-phosphate. Abbreviated names are used for some of the monosaccharides. Full names of these are given in Supplementary_data.xlsx:Worksheet4.

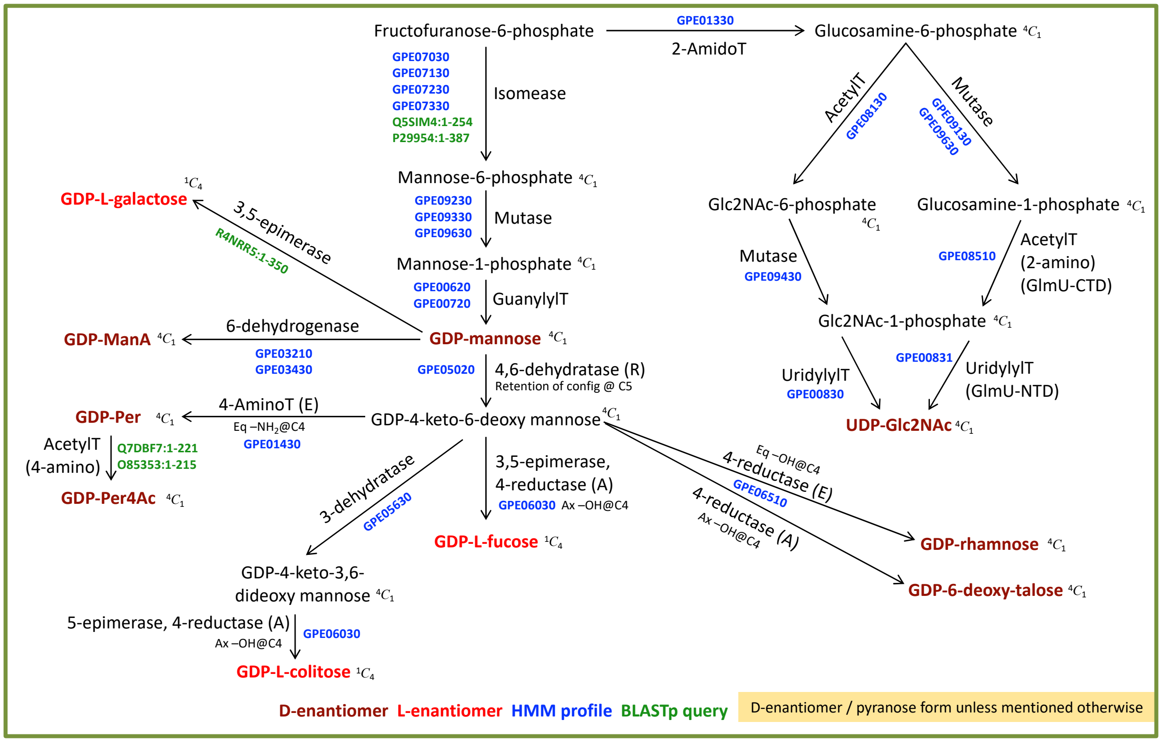

Figure S2d GDP- and UDP-linked monosaccharides derived from fructofuranose-6-phosphate. Abbreviated names are used for some of the monosaccharides. Full names of these are given in Supplementary_data.xlsx:Worksheet4.

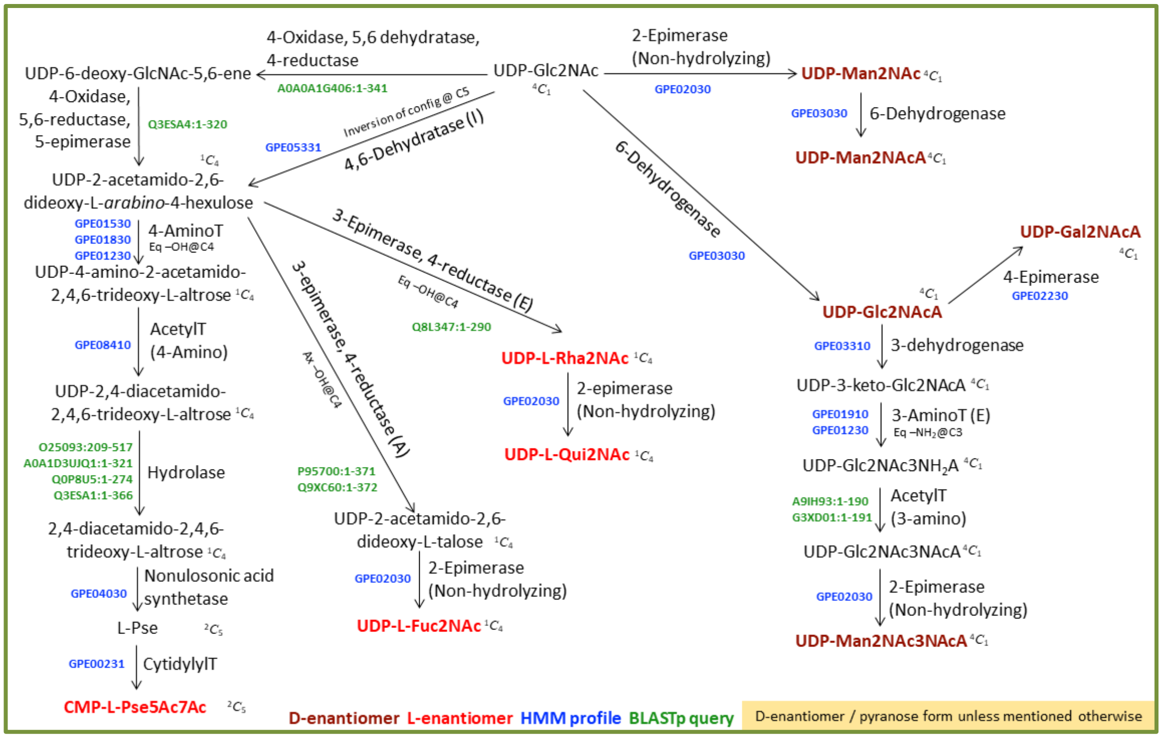

Figure S2e CMP- and UDP-linked monosaccharides derived from UDP-Glc2NAc (1 of 2). Abbreviated names are used for some of the monosaccharides. Full names of these are given in Supplementary_data.xlsx:Worksheet4.

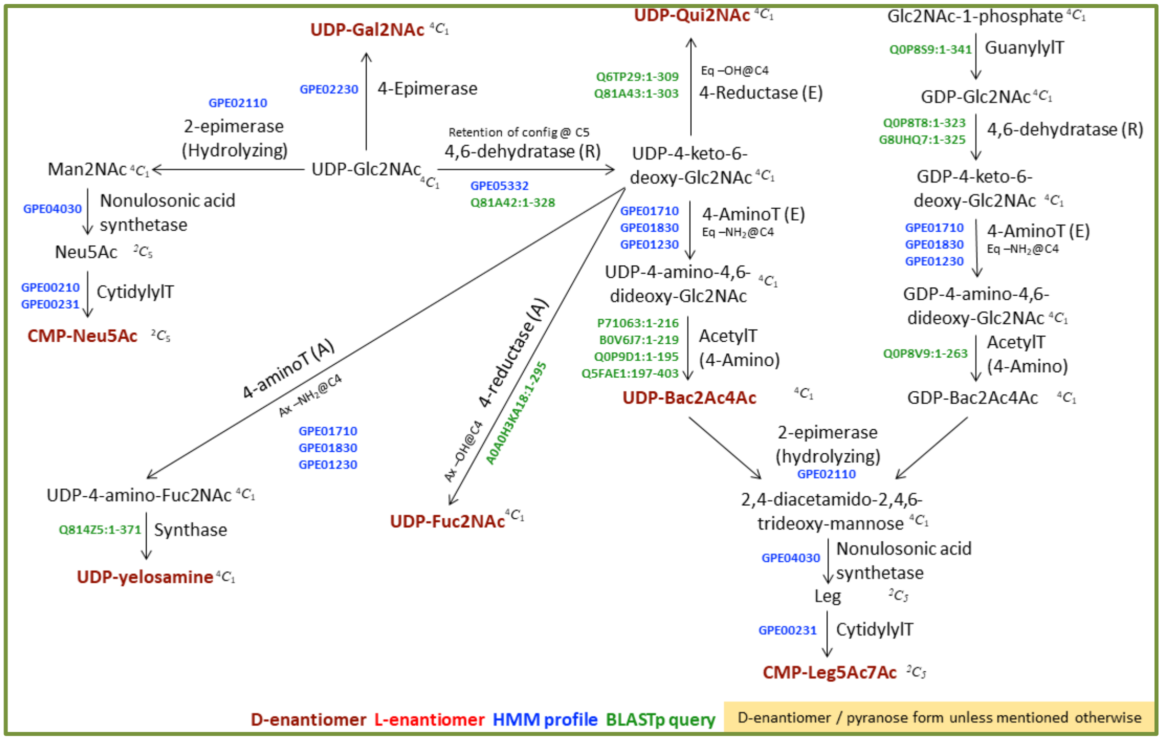

Figure S2f CMP- and UDP-linked monosaccharides derived from UDP-Glc2NAc (2 of 2). CMP-Leg5Ac7Ac may be biosynthesized through GDP-linked or UDP-linked intermediates. Abbreviated names are used for some of the monosaccharides. Full names of these are given in Supplementary_data.xlsx:Worksheet4.

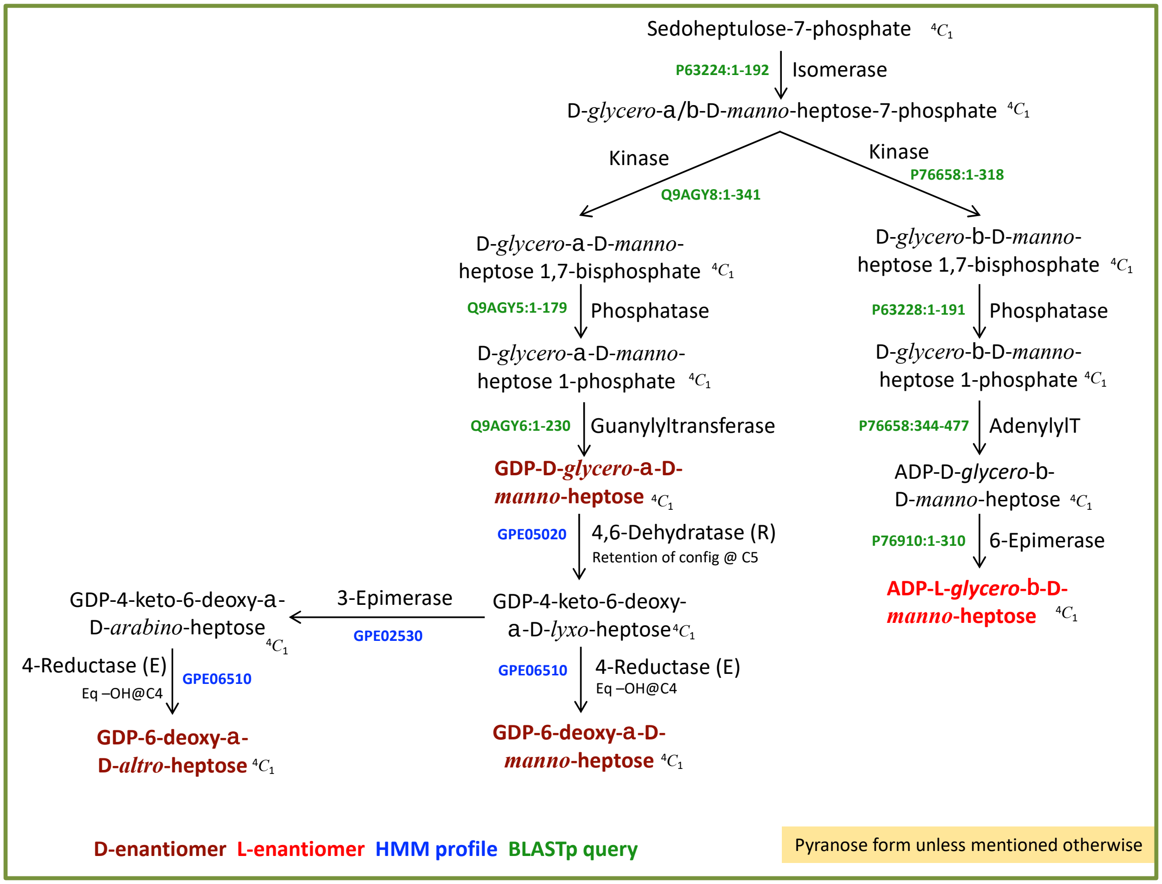

Figure S2g ADP- and GDP-linked heptoses derived from sedoheptulose-7-phosphate. Abbreviated names are used for some of the monosaccharides. Full names of these are given in Supplementary_data.xlsx:Worksheet4.

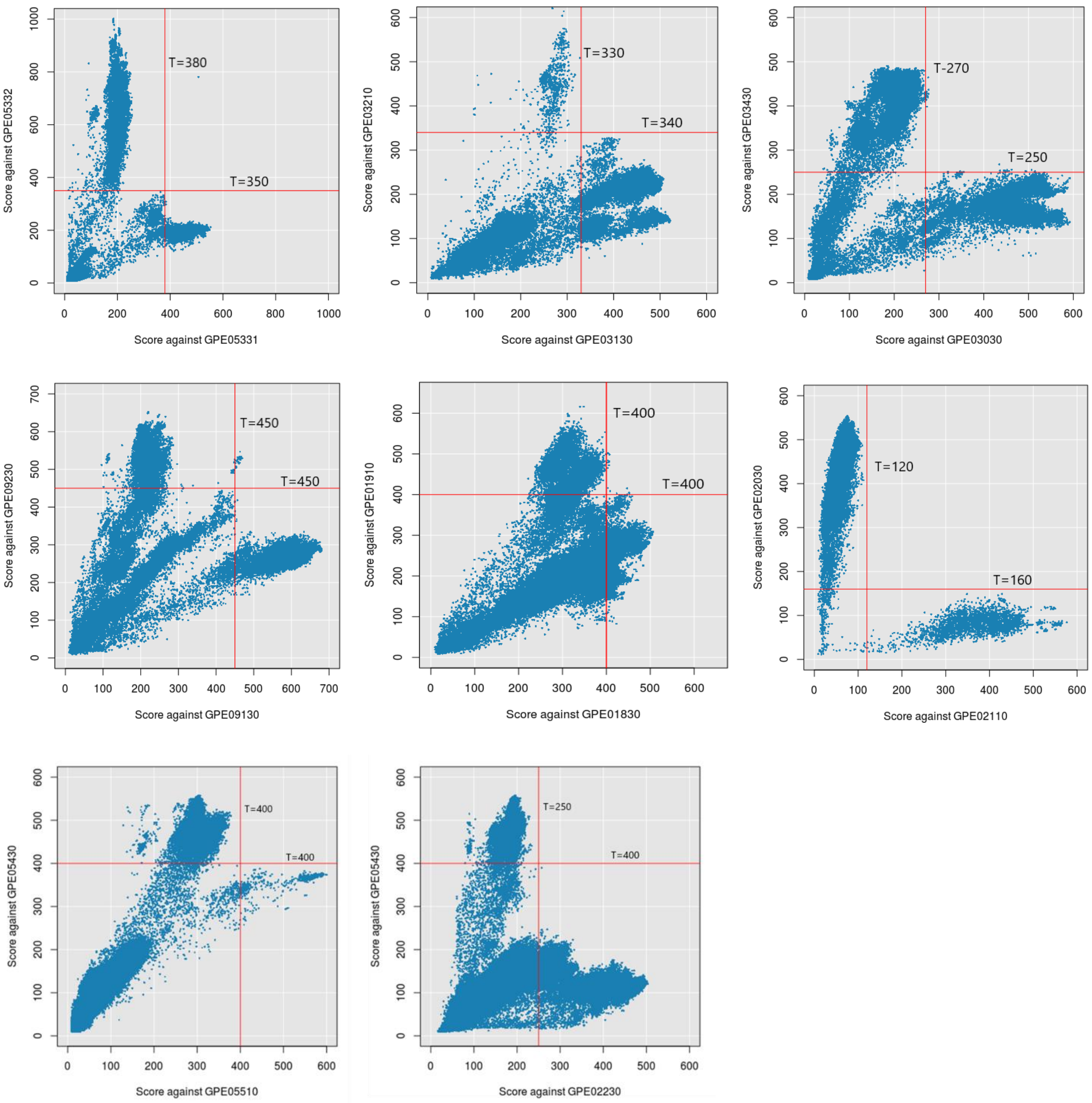

Figure S3 Setting bit score thresholds for HMM profiles with varying substrate specificities. TrEMBL database was scanned using the profiles shown along the X- and Y-axes in the above scatter plots; for these scans, default values set by HMMer were used for all the parameters. Hits that are common to a pair of profiles (shown along X- and Y-axes) were chosen and bit scores of such hits were plotted against each other. Bit score thresholds (indicated by red lines) were chosen such that a protein is a hit for only one of the two profiles. Threshold was revised for GPE05331 set to exclude PdeG.

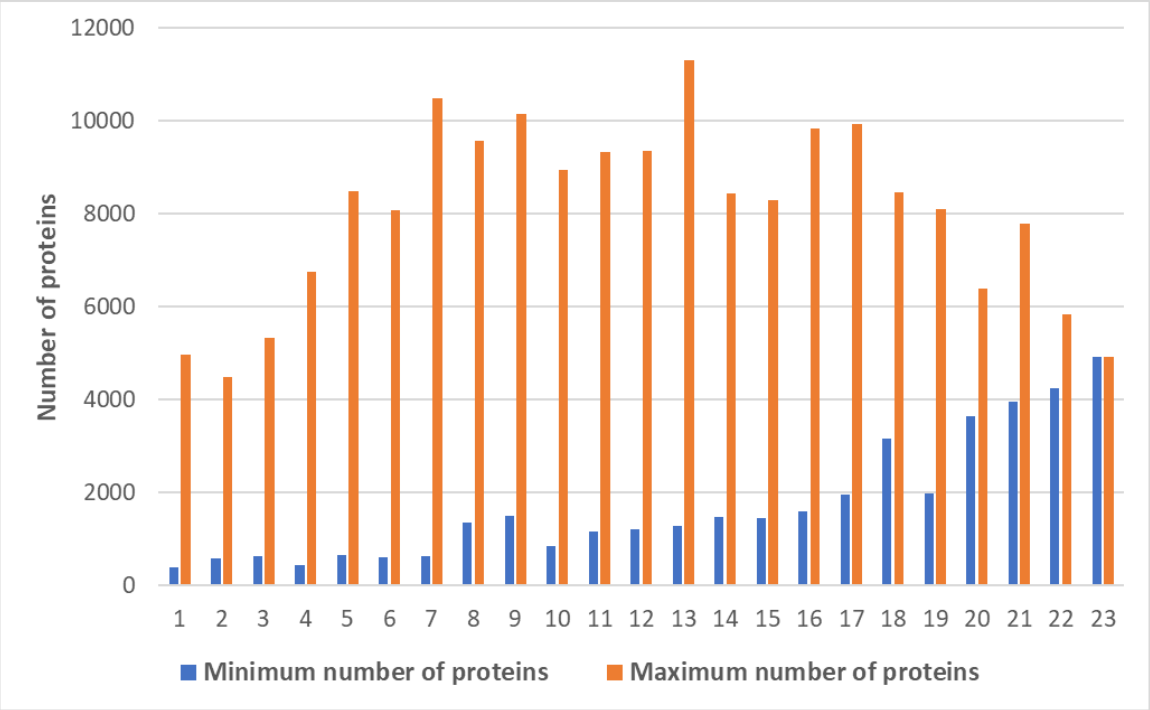

Figure S4 Variations in the proteome size of organisms which encode the same number of monosaccharides. Only the smallest and largest proteome sizes are shown. As can be seen, the number of monosaccharides used by an organism is independent of the proteome size. For instance, *Helicobacter pylori* PNG84A (proteome size = 1353) uses the same number of monosaccharides (7) as *Sorangium cellulosum* So0157-2 (proteome size = 10480).

A

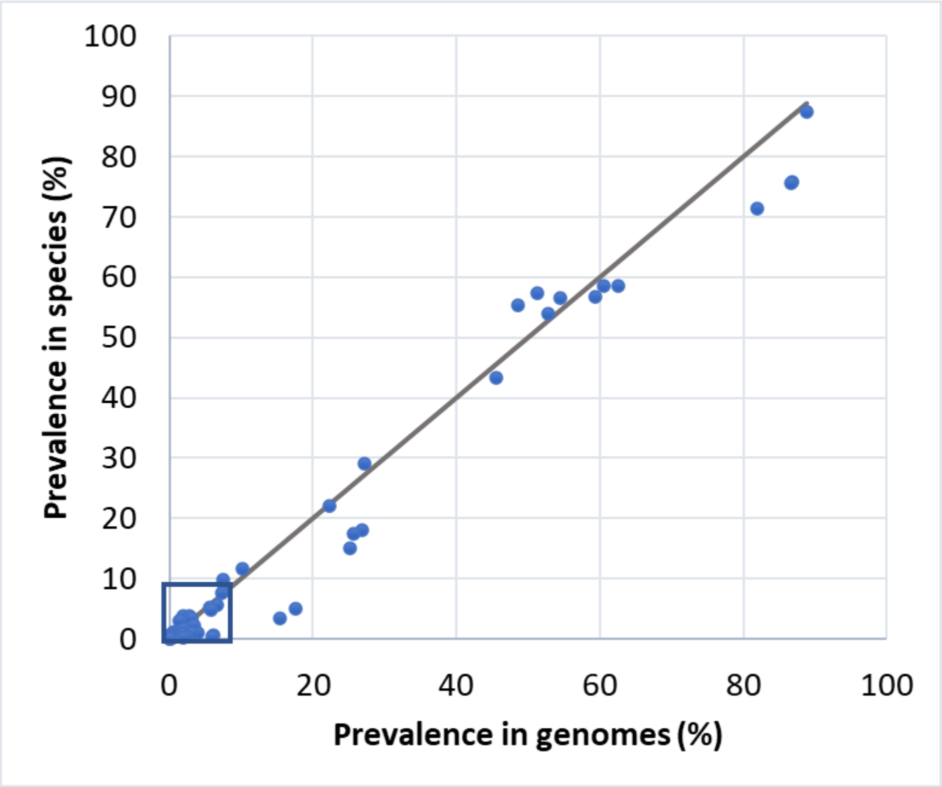
Prevalence in species (%)
Prevalence in genomes (%)
0
10
20
30
40
50
60
70
80
90
100
0
20
40
60
80
100

B

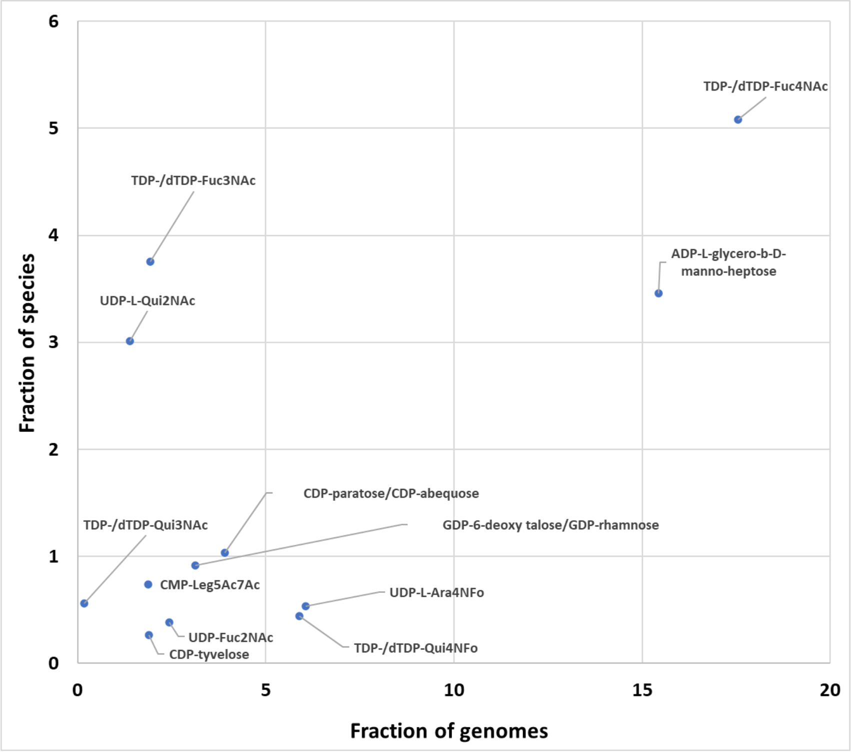
Fraction of species
Fraction of genomes
TDP-/dTDP-Fuc4NAc
TDP-/dTDP-Fuc3NAc
ADP-L-glycero-b-D-manno-heptose
UDP-L-Qui2NAc
CDP-paratose/CDP-abequose
GDP-6-deoxy talose/GDP-rhamnose
TDP-/dTDP-Qui3NAc
CMP-Leg5Ac7Ac
UDP-L-Ara4NFo
UDP-Fuc2NAc
CDP-tyvelose
TDP-/dTDP-Qui4NFo
0
1
2
3
4
5
6
0
5
10
15
20

Figure S5 (A) The prevalence of each monosaccharide as percentages of the genomes analyzed in this study (viz., 12939) and the number of species covered by these genomes (viz., 3384; Figure S1(a)). The diagonal line is manually drawn to facilitate visualization of deviations. (B) Zoomed in view of the region near the origin in (A). Data for most of the monosaccharides lie on the diagonal suggesting that the sequencing of a large number of strains for a few species has not biased the outcome, with the exception of TDP-/dTDP-Fuc4NAc and UDP-L-Qui2NAc. TDP-/dTDP-Fuc4NAc (a point below the diagonal line) is present in fewer species but represents a larger fraction of genomes since 679 strains of *E. coli* contain this monosaccharide. Conversely, presence of UDP-L-Qui2NAc (a point above the diagonal line) is highly strain specific. Abbreviated names are used for some of the monosaccharides. Full names of these are given in Supplementary_data.xlsx:Worksheet4.

Table S1 Tools and databases used in this study

Tool / Database	Version / Release	URL	Reference
Tools installed and run locally on a Linux platform			
BLASTp	2.2.31+	ftp://ftp.ncbi.nlm.nih.gov/blast/executables/blast+/2.2.31/	(1)
HMMER	3.1b2	http://hmmer.org/download.html	(2)
MUSCLE	3.8.31	https://www.ebi.ac.uk/Tools/msa/muscle/	(3)
CD-Hit	4.6	http://weizhongli-lab.org/cd-hit/	(4)
Directly accessed from the website or FTP site			
UniProt	2018_07	https://www.uniprot.org/	(5)
Genome	2019_03	https://www.ncbi.nlm.nih.gov/genome/	(6)
Pubmed	Not applicable	https://www.ncbi.nlm.nih.gov/pubmed/	(6)
CATH-Plus	4.2	http://www.cathdb.info/	(7)
PDB	Not applicable	https://www.rcsb.org/	(8)
Used through the TrEMBL database			
UniRule	Not applicable	https://www.uniprot.org/help/unirule	(5)
SAAS	Not applicable	https://www.uniprot.org/help/saas	(5)

References cited in Table S1

1. Altschul SF, Gish W, Miller W, Myers EW, Lipman DJ. Basic local alignment search tool. J Mol Biol. 1990 Oct 5;215(3):403–10.

2. Eddy SR. Accelerated Profile HMM Searches. PLoS Comput Biol. 2011 Oct;7(10):e1002195.

3. Madeira F, Park YM, Lee J, Buso N, Gur T, Madhusoodanan N, et al. The EMBL-EBI search and sequence analysis tools APIs in 2019. Nucleic Acids Res. 2019 Jul 2;47(W1):W636–41.

4. Huang Y, Niu B, Gao Y, Fu L, Li W. CD-HIT Suite: a web server for clustering and comparing biological sequences. Bioinformatics. 2010 Mar 1;26(5):680–2.

5. UniProt Consortium. UniProt: a worldwide hub of protein knowledge. Nucleic Acids Res. 2019 Jan 8;47(D1):D506–15.

6. NCBI Resource Coordinators. Database resources of the National Center for Biotechnology Information. Nucleic Acids Res. 2018 04;46(D1):D8–13.

7. Knudsen M, Wiuf C. The CATH database. Human Genomics. 2010;4(3):207.

8. Berman HM, Westbrook J, Feng Z, Gilliland G, Bhat TN, Weissig H, et al. The Protein Data Bank. Nucleic Acids Res. 2000 Jan 1;28(1):235–42.

Table S2 Monosaccharides whose both enantiomers are considered in the present study: comparison of the nucleotide to which the enantiomer is linked and the precursor for its biosynthesis

Monosaccharide	D enantiomer		L enantiomer	
	Nucleotide	Precursor	Nucleotide	Precursor
Rhamnose	GDP	Glc-1-P	TDP, dTDP, UDP	Glc-1-P
6-Deoxytalose	GDP	Glc-1-P	TDP, dTDP	Glc-1-P
Galactose	UDP	Glc-1-P	GDP	Glc-1-P
Fucose	TDP, dTDP	Glc-1-P	GDP	Glc-1-P
Fuc2NAc	UDP	UDP-Glc2NAc	UDP	UDP-Glc2NAc
Qui2NAc	UDP	UDP-Glc2NAc	UDP	UDP-Glc2NAc

Flowchart S1 Procedure used to generate HMM profiles

1. Generation of Exp dataset and Exp profile, and setting T_{exp}

Step 1a	Consider only those enzymes which are characterized by direct enzyme activity assay
Step 1b	Remove redundancy (80% sequence identity cutoff) and obtain a multiple sequence alignment (MSA)
Step 1c	Use the MSA as input to generate an HMM profile
Step 1d	Score Exp dataset sequences against this HMM profile
Step 1e	Set the bit score of the lowest scoring sequence as the bit score threshold for Exp dataset, T_{exp}

2. Generation of Extend dataset and Extend profile, and setting T_{extend}

Step 2a	Add sequences that meet any of the following criteria to the Exp dataset
(i)	SwissProt entries satisfying the threshold T_{exp}
(ii)	SwissProt entries scoring $< T_{exp}$ provided they show conservation of active site residues. Active site residues were collated based on site directed mutagenesis studies or ligand-bound 3D structures
(iii)	TrEMBL entries for which molecular function has been inferred from experiments other than direct enzyme assays viz., complementation assays, phenotypic studies, etc.
(iv)	TrEMBL entries with solved 3D structure
(v)	FunFam members (CATH database) but only in the case of CDP-glucose 4,6-dehydratase (FunFam 20603) and phosphomannoisomerase family 3 (FunFam 54112)
Step 2b	Remove redundancy (80% sequence identity cutoff) and obtain a multiple sequence alignment (MSA)
Step 2c	Use the MSA as input to generate an HMM profile
Step 2d	Score Extend dataset sequences against this HMM profile
Step 2e threshold	Set the bit score of the lowest scoring sequence as the bit score for Extend dataset, T_{extend}

Flowchart S2 Precedence rules for assigning annotation to proteins that are hits to two or more profiles and/or BLASTp queries

```
# Case 1 of 14
# specific_aminoTs = [GPE01710, GPE01430, GPE01530]
# C3_C4_aminoTs = [GPE01910, GPE01830]
# EXPECTED: for a protein which is a hit for one of the specific_aminoTs is expected
   to be a hit in C3_C4_aminoTs as well as GPE01230

    IF (hit for any one of specific_aminoTs) THEN
       IF (hit for any one of C3_C4_aminoTs) THEN
           IF (hit for GPE01230) THEN
               Pass (i.e., this is as expected)
           ELSE
               Alert: Hit for one of C3_C4_aminoTs but not GPE01230
           ENDIF
       ELSE
           Alert: Hit for one of specific_aminoTs but not C3_C4_aminoTs
       ENDIF
    ENDIF
    IF (hit for any one of C3_C4_aminoTs) THEN
       IF (hit for GPE01230) THEN
           Pass (as expected)
       ELSE
           Alert: Hit for one of C3_C4_aminoTs but not GPE01230
       ENDIF
   ENDIF
```

```
# Case 2 of 14
    IF a protein is a hit for any one of specific_aminoTs, it should be assigned that
    annotation
         IF (hit for any one of specific_aminoTs) THEN
           Assign annotation
         ENDIF
    ENDIF
```

```
# Case 3 of 14
# For a protein which is a hit for one of C3_C4_aminoTs but not any of
specific_aminoTs, it should be assigned the former
    IF (hit for one of C3_C4_aminoTs and not for any of specific_aminoTs) THEN
       Assign GPE01830/GPE01910 annotation
    ENDIF
```

```
# Case 4 of 14
# For a protein which is a hit for GPE00210
# GPE00210 is used only in combination with GPE00231
# A protein which is a hit for GPE00210 is expected to be a hit for GPE00231 also
    IF (hit for GPE00210) THEN
      IF (hit for GPE00231) THEN
         Assign GPE00210 annotation
      ELSE
         Alert: Hit for GPE00210 but not GPE00231
      ENDIF
    ENDIF
```

```
# Case 5 of 14
# For a protein which is a hit for GPE02430
# GPE02430 is used only in combination with GPE02530
# A protein which is a hit for GPE02430 is expected to be a hit for GPE02530 also
    IF (hit for GPE02430) THEN
      IF (hit for GPE002530) THEN
         Assign GPE02430 annotation
      ELSE
         Alert: Hit for GPE02430 but not GPE02530
      ENDIF
    ENDIF
```

```
# Case 6 of 14
# For a protein that is a hit for GPE03130
# GPE03130 is used only in combination with GPE03430
# A protein which is a hit for GPE03130 is expected to be a hit for GPE03430 also
    IF (hit for GPE03130) THEN
      IF (hit for GPE03430) THEN
         Assign GPE03130 annotation
      ELSE
         Alert: Hit for GPE03130 but not GPE03430
      ENDIF
    ENDIF
```

```
# Case 7 of 14
# For a protein that is a hit for GPE03210
# GPE03210 is used only in combination with GPE03430
# A protein which is a hit for GPE03210 is expected to be a hit for GPE03430 also
    IF (hit for GPE03210) THEN
      IF (hit for GPE03430) THEN
          Assign GPE03210 annotation
      ELSE
          Alert: Hit for GPE03210 but not GPE03430
      ENDIF
    ENDIF
```

```
# Case 8 of 14
# For a protein that is a hit for GPE09130
# GPE09130 is used only in combination with GPE09630
# A protein which is a hit for GPE09130 is expected to be a hit for GPE09630 also
    IF (hit for GPE09130) THEN
      IF (hit for GPE09630) THEN
          Assign GPE09130 annotation
      ELSE
          Alert: Hit for GPE09130 but not GPE09630
      ENDIF
    ENDIF
```

```
# Case 9 of 14
# For a protein that is a hit for GPE09230
# GPE09230 is used only in combination with GPE09330 and GPE09630
# A protein which is a hit for GPE09230 is expected to be a hit for GPE09630 also
    IF (hit for GPE09230) THEN
      IF (hit for GPE09630) THEN
          Assign GPE09230 annotation
      ELSE
          Alert: Hit for GPE09230 but not GPE09630
      ENDIF
    ENDIF
```

```
# Case 10 of 14
# For a protein that is a hit for GPE09330 and GPE09630
# GPE09330 is used only in combination with GPE09630
# A protein can be a hit for GPE09330 or GPE09630, but not for both (non-
  orthologous)
    IF (hit for GPE09330 AND hit for GPE09630) THEN
        Alert: Hit for GPE09330 and GPE09630
    ENDIF
```

```
# Case 11 of 14
# For a protein that is a hit for GPE00620 and GPE00720
# GPE00620 is used only in combination with GPE00720
# A protein can be a hit for GPE00620 or GPE00720, but not for both (non-
  orthologous)
    IF (hit for GPE00620 AND hit for GPE00720) THEN
        Alert: Hit for GPE00620 and GPE00720
    ENDIF
```

```
# Case 12 of 14
# Isomerases: GPE07030, GPE07130, GPE07230, and GPE07330
# A protein can be a hit for any one of the above four profiles (non-orthologous)
      For GPE07030, GPE07130, GPE07230 and GPE07330
      IF (hit for more than one)
           Alert: Hit for (list all profiles which appear as hits from above list)]
```

```
# Case 13 of 14
# For a protein that is a hit for GPE00430 and GPE00530
# GPE00430 is used only in combination with GPE00530
# A protein can be a hit for GPE00430 or GPE00530, but not for both (non-
  orthologous)
    IF (hit for GPE00430 AND hit for GPE00530) THEN
        Alert: Hit for GPE00430 and GPE00530
    ENDIF
```

```
# Case 14 of 14
# For a protein that is a hit for GPE05332 and Q81A42:1-328
# GPE05332 is used only in combination with Q81A42:1-328
# A protein can be a hit for GPE05332 or Q81A42:1-328, but not for both (non-
  orthologous)
    IF (hit for GPE05332 AND hit for Q81A42:1-328) THEN
        Alert: Hit for GPE05332 and Q81A42:1-328
    ENDIF
```

116

Research articles which report the characterization of enzymes involved in the biosynthesis of monosaccharides are listed below. Amino acid sequences of these enzymes were either used to generate HMM profiles or used as BLASTp queries. The PubMed Ids of these research articles are included in the GlycoPathDB (www.bio.iitb.ac.in/glycopathdb/) against respective sequence entry. These PubMed Ids are hyperlinked to the corresponding PubMed webpage.

1. Wang-Gillam A, Pastuszak I, Elbein AD. A 17-amino acid insert changes UDP-N-acetylhexosamine pyrophosphorylase specificity from UDP-GalNAc to UDP-GlcNAc. J Biol Chem. 1998 Oct 16;273(42):27055–7.

2. Watt G, Leoff C, Harper AD, Bar-Peled M. A bifunctional 3,5-epimerase/4-keto reductase for nucleotide-rhamnose synthesis in Arabidopsis. Plant Physiol. 2004 Apr;134(4):1337–46.

3. Hinderlich S, Stäsche R, Zeitler R, Reutter W. A bifunctional enzyme catalyzes the first two steps in N-acetylneuraminic acid biosynthesis of rat liver. Purification and characterization of. J Biol Chem. 1997 Sep 26;272(39):24313–8.

4. Breazeale SD, Ribeiro AA, McClerren AL, Raetz CRH. A formyltransferase required for polymyxin resistance in Escherichia coli and the modification of lipid A with 4-Amino-4-deoxy-L-arabinose. Identification and function oF UDP-4-deoxy-4-formamido-L-arabinose. J Biol Chem. 2005 Apr 8;280(14):14154–67.

5. Yoo H-G, Kwon S-Y, Karki S, Kwon H-J. A new route to dTDP-6-deoxy-l-talose and dTDP-L-rhamnose: dTDP-L-rhamnose. Bioorg Med Chem Lett. 2011 Jul 1;21(13):3914–7.

6. Yoshida Y, Nakano Y, Nezu T, Yamashita Y, Koga T. A novel NDP-6-deoxyhexosyl-4-ulose reductase in the pathway for the synthesis of thymidine diphosphate-D-fucose. J Biol Chem. 1999 Jun 11;274(24):16933–9.

7. Swan MK, Hansen T, Schönheit P, Davies C. A novel phosphoglucose isomerase (PGI)/phosphomannose isomerase from the crenarchaeon Pyrobaculum aerophilum is a member of the PGI superfamily: structural evidence at 1.16-A resolution. J Biol Chem. 2004 Sep 17;279(38):39838–45.

8. Jiang H, Wang S, Dang L, Wang S, Chen H, Wu Y, et al. A novel short-root gene encodes a glucosamine-6-phosphate acetyltransferase required for maintaining normal root cell shape in rice. Plant Physiol. 2005 May;138(1):232–42.

9. DeHaven JE, Robinson KA, Nelson BA, Buse MG. A novel variant of glutamine: fructose-6-phosphate amidotransferase-1 (GFAT1) mRNA is selectively expressed in striated muscle. Diabetes. 2001 Nov;50(11):2419–24.

10. Jia X, Kang J, Yin H. A simple and rapid method for measuring α-D-phosphohexomutases activity by using anion-exchange chromatography coupled

with an electrochemical detector. PeerJ. 2016;4:e1517.

11. Bernatchez S, Szymanski CM, Ishiyama N, Li J, Jarrell HC, Lau PC, et al. A single bifunctional UDP-GlcNAc/Glc 4-epimerase supports the synthesis of three cell surface glycoconjugates in Campylobacter jejuni. J Biol Chem. 2005 Feb 11;280(6):4792–802.

12. Velloso LM, Bhaskaran SS, Schuch R, Fischetti VA, Stebbins CE. A structural basis for the allosteric regulation of non-hydrolysing UDP-GlcNAc. EMBO Rep. 2008 Feb;9(2):199–205.

13. Cook PD, Carney AE, Holden HM. Accommodation of GDP-linked sugars in the active site of GDP-perosamine synthase. Biochemistry. 2008 Oct 7;47(40):10685–93.

14. Namboori SC, Graham DE. Acetamido sugar biosynthesis in the Euryarchaea. J Bacteriol. 2008 Apr;190(8):2987–96.

15. Gehring AM, Lees WJ, Mindiola DJ, Walsh CT, Brown ED. Acetyltransfer precedes uridylyltransfer in the formation of UDP-N-acetylglucosamine in separable active sites of the bifunctional GlmU protein of Escherichia coli. Biochemistry. 1996 Jan 16;35(2):579–85.

16. Thoden JB, Holden HM. Active site geometry of glucose-1-phosphate uridylyltransferase. Protein Sci. 2007 Jul;16(7):1379–88.

17. Samuel J, Tanner ME. Active site mutants of the “non-hydrolyzing” UDP-N-acetylglucosamine 2-epimerase from Escherichia coli. Biochim Biophys Acta. 2004 Jul 1;1700(1):85–91.

18. Rashid N, Kanai T, Atomi H, Imanaka T. Among multiple phosphomannomutase gene orthologues, only one gene encodes a protein with phosphoglucomutase and phosphomannomutase activities in Thermococcus kodakaraensis. J Bacteriol. 2004 Sep;186(18):6070–6.

19. Qu H, Xin Y, Dong X, Ma Y. An rmlA gene encoding d-glucose-1-phosphate thymidylyltransferase is essential for mycobacterial growth. FEMS Microbiol Lett. 2007 Oct;275(2):237–43.

20. Merson-Davies LA, Cundliffe E. Analysis of five tylosin biosynthetic genes from the tyllBA region of the Streptomyces fradiae genome. Mol Microbiol. 1994 Jul;13(2):349–55.

21. Li W, Ulm H, Rausch M, Li X, O’Riordan K, Lee JC, et al. Analysis of the Staphylococcus aureus capsule biosynthesis pathway in vitro: characterization of the UDP-GlcNAc C6 dehydratases CapD and CapE and identification of enzyme inhibitors. Int J Med Microbiol. 2014 Nov;304(8):958–69.

22. Maruta T, Yonemitsu M, Yabuta Y, Tamoi M, Ishikawa T, Shigeoka S. Arabidopsis phosphomannose isomerase 1, but not phosphomannose isomerase 2, is essential for ascorbic acid biosynthesis. J Biol Chem. 2008 Oct 24;283(43):28842–51.

23. Bahat-Samet E, Castro-Sowinski S, Okon Y. Arabinose content of extracellular polysaccharide plays a role in cell aggregation of Azospirillum brasilense. FEMS Microbiol Lett. 2004 Aug 15;237(2):195–203.

24. Grangeasse C, Obadia B, Mijakovic I, Deutscher J, Cozzone AJ, Doublet P. Autophosphorylation of the Escherichia coli protein kinase Wzc regulates tyrosine phosphorylation of Ugd, a UDP-glucose dehydrogenase. J Biol Chem. 2003 Oct 10;278(41):39323–9.

25. Sandlin RC, Lampel KA, Keasler SP, Goldberg MB, Stolzer AL, Maurelli AT. Avirulence of rough mutants of Shigella flexneri: requirement of O antigen for correct unipolar localization of IcsA in the bacterial outer membrane. Infect Immun. 1995 Jan;63(1):229–37.

26. Kotake T, Takata R, Verma R, Takaba M, Yamaguchi D, Orita T, et al. Bifunctional cytosolic UDP-glucose 4-epimerases catalyse the interconversion between. Biochem J. 2009 Nov 11;424(2):169–77.

27. Hansen T, Wendorff D, Schönheit P. Bifunctional phosphoglucose/phosphomannose isomerases from the Archaea Aeropyrum pernix and Thermoplasma acidophilum constitute a novel enzyme family within the phosphoglucose isomerase superfamily. J Biol Chem. 2004 Jan 16;279(3):2262–72.

28. Wu B, Zhang Y, Zheng R, Guo C, Wang PG. Bifunctional phosphomannose isomerase/GDP-D-mannose pyrophosphorylase is the point of control for GDP-D-mannose biosynthesis in Helicobacter pylori. FEBS Lett. 2002 May 22;519(1–3):87–92.

29. Morrison MJ, Imperiali B. Biochemical analysis and structure determination of bacterial acetyltransferases responsible for the biosynthesis of UDP-N,N'-diacetylbacillosamine. J Biol Chem. 2013 Nov 8;288(45):32248–60.

30. Mao W, Daligaux P, Lazar N, Ha-Duong T, Cavé C, van Tilbeurgh H, et al. Biochemical analysis of leishmanial and human GDP-Mannose Pyrophosphorylases and selection of inhibitors as new leads. Sci Rep. 2017 Apr 7;7(1):751.

31. Sousa SA, Feliciano JR, Pinheiro PF, Leitão JH. Biochemical and functional studies on the Burkholderia cepacia complex bceN gene, encoding a GDP-D-mannose 4,6-dehydratase. PLoS One. 2013;8(2):e56902.

32. Thoden JB, Holden HM. Biochemical and structural characterization of WlbA

from Bordetella pertussis and Chromobacterium violaceum: enzymes required for the biosynthesis of 2,3-diacetamido-2,3-dideoxy-D-mannuronic acid. Biochemistry. 2011 Mar 8;50(9):1483–91.

33. Granja AT, Popescu A, Marques AR, Sá-Correia I, Fialho AM. Biochemical characterization and phylogenetic analysis of UDP-glucose dehydrogenase from the gellan gum producer Sphingomonas elodea ATCC 31461. Appl Microbiol Biotechnol. 2007 Oct;76(6):1319–27.

34. Wang Y, Xu Y, Perepelov AV, Qi Y, Knirel YA, Wang L, et al. Biochemical characterization of dTDP-D-Qui4N and dTDP-D-Qui4NAc biosynthetic pathways in Shigella dysenteriae type 7 and Escherichia coli O7. J Bacteriol. 2007 Dec;189(23):8626–35.

35. Ren Y, Perepelov AV, Wang H, Zhang H, Knirel YA, Wang L, et al. Biochemical characterization of GDP-L-fucose de novo synthesis pathway in fungus Mortierella alpina. Biochem Biophys Res Commun. 2010 Jan 22;391(4):1663–9.

36. Hartley MD, Morrison MJ, Aas FE, Børud B, Koomey M, Imperiali B. Biochemical characterization of the O-linked glycosylation pathway in Neisseria gonorrhoeae responsible for biosynthesis of protein glycans containing N,N’-diacetylbacillosamine. Biochemistry. 2011 Jun 7;50(22):4936–48.

37. Guo H, Li L, Wang PG. Biochemical characterization of UDP-GlcNAc/Glc 4-epimerase from Escherichia coli O86:B7. Biochemistry. 2006 Nov 21;45(46):13760–8.

38. Riegert AS, Chantigian DP, Thoden JB, Tipton PA, Holden HM. Biochemical Characterization of WbkC, an N-Formyltransferase from Brucella melitensis. Biochemistry. 2017 Jul 18;56(28):3657–68.

39. Miller WL, Wenzel CQ, Daniels C, Larocque S, Brisson J-R, Lam JS. Biochemical characterization of WbpA, a UDP-N-acetyl-D-glucosamine 6-dehydrogenase involved in O-antigen biosynthesis in Pseudomonas aeruginosa PAO1. J Biol Chem. 2004 Sep 3;279(36):37551–8.

40. Dunsirn MM, Thoden JB, Gilbert M, Holden HM. Biochemical Investigation of Rv3404c from Mycobacterium tuberculosis. Biochemistry. 2017 Jul 25;56(29):3818–25.

41. Salinger AJ, Brown HA, Thoden JB, Holden HM. Biochemical studies on WbcA, a sugar epimerase from Yersinia enterocolitica. Protein Sci. 2015 Oct;24(10):1633–9.

42. Tsukioka Y, Yamashita Y, Oho T, Nakano Y, Koga T. Biological function of the dTDP-rhamnose synthesis pathway in Streptococcus mutans. J Bacteriol. 1997 Feb;179(4):1126–34.

43. Kneidinger B, Larocque S, Brisson J-R, Cadotte N, Lam JS. Biosynthesis of 2-acetamido-2,6-dideoxy-L-hexoses in bacteria follows a pattern distinct from those of the pathways of 6-deoxy-L-hexoses. Biochem J. 2003 May 1;371(Pt 3):989–95.

44. Glaze PA, Watson DC, Young NM, Tanner ME. Biosynthesis of CMP-N,N'-diacetyllegionaminic acid from. Biochemistry. 2008 Mar 11;47(10):3272–82.

45. Alam J, Beyer N, Liu H. Biosynthesis of colitose: expression, purification, and mechanistic characterization of GDP-4-keto-6-deoxy-D-mannose-3-dehydrase (ColD) and GDP-L-colitose synthase (ColC). Biochemistry. 2004 Dec 28;43(51):16450–60.

46. Pfoestl A, Hofinger A, Kosma P, Messner P. Biosynthesis of dTDP-3-acetamido-3,6-dideoxy-alpha-D-galactose in Aneurinibacillus thermoaerophilus L420-91T. J Biol Chem. 2003 Jul 18;278(29):26410–7.

47. Sanz S, Bandini G, Ospina D, Bernabeu M, Mariño K, Fernández-Becerra C, et al. Biosynthesis of GDP-fucose and other sugar nucleotides in the blood stages of Plasmodium falciparum. J Biol Chem. 2013 Jun 7;288(23):16506–17.

48. Kneidinger B, Graninger M, Puchberger M, Kosma P, Messner P. Biosynthesis of nucleotide-activated D-glycero-D-manno-heptose. J Biol Chem. 2001 Jun 15;276(24):20935–44.

49. Martinez V, Ingwers M, Smith J, Glushka J, Yang T, Bar-Peled M. Biosynthesis of UDP-4-keto-6-deoxyglucose and UDP-rhamnose in pathogenic fungi Magnaporthe grisea and Botryotinia fuckeliana. J Biol Chem. 2012 Jan 6;287(2):879–92.

50. Larkin A, Imperiali B. Biosynthesis of UDP-GlcNAc(3NAc)A by WbpB, WbpE, and WbpD: enzymes in the Wbp pathway responsible for O-antigen assembly in Pseudomonas aeruginosa PAO1. Biochemistry. 2009 Jun 16;48(23):5446–55.

51. Broach B, Gu X, Bar-Peled M. Biosynthesis of UDP-glucuronic acid and UDP-galacturonic acid in Bacillus cereus subsp. cytotoxis NVH 391-98. FEBS J. 2012 Jan;279(1):100–12.

52. Mulrooney EF, Poon KKH, McNally DJ, Brisson J-R, Lam JS. Biosynthesis of UDP-N-acetyl-L-fucosamine, a precursor to the biosynthesis of lipopolysaccharide in Pseudomonas aeruginosa serotype O11. J Biol Chem. 2005 May 20;280(20):19535–42.

53. Morrison MJ, Imperiali B. Biosynthesis of UDP-N,N'-diacetylbacillosamine in Acinetobacter baumannii: Biochemical characterization and correlation to existing pathways. Arch Biochem Biophys. 2013 Aug 1;536(1):72–80.

54. Gu X, Lee SG, Bar-Peled M. Biosynthesis of UDP-xylose and UDP-arabinose in Sinorhizobium meliloti 1021: first characterization of a bacterial UDP-xylose

synthase, and UDP-xylose 4-epimerase. Microbiology. 2011 Jan;157(Pt 1):260–9.

55. Harper AD, Bar-Peled M. Biosynthesis of UDP-xylose. Cloning and characterization of a novel Arabidopsis gene family, UXS, encoding soluble and putative membrane-bound UDP-glucuronic acid decarboxylase isoforms. Plant Physiol. 2002 Dec;130(4):2188–98.

56. Kneidinger B, Marolda C, Graninger M, Zamyatina A, McArthur F, Kosma P, et al. Biosynthesis pathway of ADP-L-glycero-beta-D-manno-heptose in Escherichia coli. J Bacteriol. 2002 Jan;184(2):363–9.

57. Weston A, Stern RJ, Lee RE, Nassau PM, Monsey D, Martin SL, et al. Biosynthetic origin of mycobacterial cell wall galactofuranosyl residues. Tuber Lung Dis. 1997;78(2):123–31.

58. Poolman B, Royer TJ, Mainzer SE, Schmidt BF. Carbohydrate utilization in Streptococcus thermophilus: characterization of the genes for aldose 1-epimerase (mutarotase) and UDPglucose 4-epimerase. J Bacteriol. 1990 Jul;172(7):4037–47.

59. Niehues R, Hasilik M, Alton G, Körner C, Schiebe-Sukumar M, Koch HG, et al. Carbohydrate-deficient glycoprotein syndrome type Ib. Phosphomannose isomerase deficiency and mannose therapy. J Clin Invest. 1998 Apr 1;101(7):1414–20.

60. Thoden JB, Reinhardt LA, Cook PD, Menden P, Cleland WW, Holden HM. Catalytic mechanism of perosamine N-acetyltransferase revealed by high-resolution. Biochemistry. 2012 Apr 24;51(16):3433–44.

61. Hwang B-Y, Lee H-J, Yang Y-H, Joo H-S, Kim B-G. Characterization and investigation of substrate specificity of the sugar aminotransferase WecE from E. coli K12. Chem Biol. 2004 Jul;11(7):915–25.

62. Soldo B, Lazarevic V, Pooley HM, Karamata D. Characterization of a Bacillus subtilis thermosensitive teichoic acid-deficient mutant: gene mnaA (yvyH) encodes the UDP-N-acetylglucosamine 2-epimerase. J Bacteriol. 2002 Aug;184(15):4316–20.

63. Piacente F, Bernardi C, Marin M, Blanc G, Abergel C, Tonetti MG. Characterization of a UDP-N-acetylglucosamine biosynthetic pathway encoded by the giant DNA virus Mimivirus. Glycobiology. 2014 Jan;24(1):51–61.

64. Haft RF, Wessels MR, Mebane MF, Conaty N, Rubens CE. Characterization of cpsF and its product CMP-N-acetylneuraminic acid synthetase, a group B streptococcal enzyme that can function in K1 capsular polysaccharide biosynthesis in Escherichia coli. Mol Microbiol. 1996 Feb;19(3):555–63.

65. Tenhaken R, Voglas E, Cock JM, Neu V, Huber CG. Characterization of GDP-mannose dehydrogenase from the brown alga Ectocarpus siliculosus providing the

precursor for the alginate polymer. J Biol Chem. 2011 May 13;286(19):16707–15.

66. Badet-Denisot MA, Fernandez-Herrero LA, Berenguer J, Ooi T, Badet B. Characterization of L-glutamine:D-fructose-6-phosphate amidotransferase from an extreme thermophile Thermus thermophilus HB8. Arch Biochem Biophys. 1997 Jan 1;337(1):129–36.

67. Sundaram AK, Pitts L, Muhammad K, Wu J, Betenbaugh M, Woodard RW, et al. Characterization of N-acetylneuraminic acid synthase isoenzyme 1 from Campylobacter jejuni. Biochem J. 2004 Oct 1;383(Pt 1):83–9.

68. Asención Diez MD, Peirú S, Demonte AM, Gramajo H, Iglesias AA. Characterization of recombinant UDP- and ADP-glucose pyrophosphorylases and glycogen synthase to elucidate glucose-1-phosphate partitioning into oligo- and polysaccharides in Streptomyces coelicolor. J Bacteriol. 2012 Mar;194(6):1485–93.

69. Mochalkin I, Lightle S, Zhu Y, Ohren JF, Spessard C, Chirgadze NY, et al. Characterization of substrate binding and catalysis in the potential antibacterial target N-acetylglucosamine-1-phosphate uridyltransferase (GlmU). Protein Sci. 2007 Dec;16(12):2657–66.

70. Melançon CE 3rd, Hong L, White JA, Liu Y, Liu H. Characterization of TDP-4-keto-6-deoxy-D-glucose-3,4-ketoisomerase from the. Biochemistry. 2007 Jan 16;46(2):577–90.

71. Cuccui J, Milne TS, Harmer N, George AJ, Harding SV, Dean RE, et al. Characterization of the Burkholderia pseudomallei K96243 capsular polysaccharide I coding region. Infect Immun. 2012 Mar;80(3):1209–21.

72. McCallum M, Shaw GS, Creuzenet C. Characterization of the dehydratase WcbK and the reductase WcaG involved in. Biochem J. 2011 Oct 15;439(2):235–48.

73. Wang Q, Ding P, Perepelov AV, Xu Y, Wang Y, Knirel YA, et al. Characterization of the dTDP-D-fucofuranose biosynthetic pathway in Escherichia coli O52. Mol Microbiol. 2008 Dec;70(6):1358–67.

74. Li ZZ, Riegert AS, Goneau M-F, Cunningham AM, Vinogradov E, Li J, et al. Characterization of the dTDP-Fuc3N and dTDP-Qui3N biosynthetic pathways in Campylobacter jejuni 81116. Glycobiology. 2017 Apr 1;27(4):358–69.

75. Macpherson DF, Manning PA, Morona R. Characterization of the dTDP-rhamnose biosynthetic genes encoded in the rfb locus of Shigella flexneri. Mol Microbiol. 1994 Jan;11(2):281–92.

76. Hofmann M, Boles E, Zimmermann FK. Characterization of the essential yeast gene encoding N-acetylglucosamine-phosphate mutase. Eur J Biochem. 1994 Apr 15;221(2):741–7.

77. Narasaki CT, Mertens K, Samuel JE. Characterization of the GDP-D-mannose biosynthesis pathway in Coxiella burnetii: the initial steps for GDP-β-D-virenose biosynthesis. PLoS One. 2011;6(10):e25514.

78. Campos M, Martínez-Salazar JM, Lloret L, Moreno S, Núñez C, Espín G, et al. Characterization of the gene coding for GDP-mannose dehydrogenase (algD) from Azotobacter vinelandii. J Bacteriol. 1996 Apr;178(7):1793–9.

79. Berg TO, Gurung MK, Altermark B, Smalås AO, Ræder ILU. Characterization of the N-acetylneuraminic acid synthase (NeuB) from the psychrophilic fish pathogen Moritella viscosa. Carbohydr Res. 2015 Jan 30;402:133–45.

80. Gurung MK, Ræder ILU, Altermark B, Smalås AO. Characterization of the sialic acid synthase from Aliivibrio salmonicida suggests a novel pathway for bacterial synthesis of 7-O-acetylated sialic acids. Glycobiology. 2013 Jul;23(7):806–19.

81. Kharel MK, Lian H, Rohr J. Characterization of the TDP-D-ravidosamine biosynthetic pathway: one-pot enzymatic synthesis of TDP-D-ravidosamine from thymidine-5-phosphate and glucose-1-phosphate. Org Biomol Chem. 2011 Mar 21;9(6):1799–808.

82. Suzuki S, Matsuzawa T, Nukigi Y, Takegawa K, Tanaka N. Characterization of two different types of UDP-glucose/-galactose 4-epimerase involved in galactosylation in fission yeast. Microbiology. 2010 Mar;156(Pt 3):708–18.

83. Mariño K, Güther MLS, Wernimont AK, Qiu W, Hui R, Ferguson MAJ. Characterization, localization, essentiality, and high-resolution crystal structure of glucosamine 6-phosphate N-acetyltransferase from Trypanosoma brucei. Eukaryot Cell. 2011 Jul;10(7):985–97.

84. Zhang L, Muthana MM, Yu H, McArthur JB, Qu J, Chen X. Characterizing non-hydrolyzing Neisseria meningitidis serogroup A. Carbohydr Res. 2016 Jan;419:18–28.

85. Partha SK, Sadeghi-Khomami A, Slowski K, Kotake T, Thomas NR, Jakeman DL, et al. Chemoenzymatic synthesis, inhibition studies, and X-ray crystallographic analysis of the phosphono analog of UDP-Galp as an inhibitor and mechanistic probe for. J Mol Biol. 2010 Nov 5;403(4):578–90.

86. Vijayakumar S, Merkx-Jacques A, Ratnayake DB, Gryski I, Obhi RK, Houle S, et al. Cj1121c, a novel UDP-4-keto-6-deoxy-GlcNAc C-4 aminotransferase essential for protein glycosylation and virulence in Campylobacter jejuni. J Biol Chem. 2006 Sep 22;281(38):27733–43.

87. Demendi M, Creuzenet C. Cj1123c (PglD), a multifaceted acetyltransferase from Campylobacter jejuni. Biochem Cell Biol. 2009 Jun;87(3):469–83.

88. Roper JR, Ferguson MAJ. Cloning and characterisation of the UDP-glucose 4’-

epimerase of Trypanosoma cruzi. Mol Biochem Parasitol. 2003 Nov;132(1):47–53.

89. Mizanur RM, Pohl NL. Cloning and characterization of a heat-stable CMP-N-acylneuraminic acid synthetase from Clostridium thermocellum. Appl Microbiol Biotechnol. 2007 Sep;76(4):827–34.

90. Zhao G, Liu J, Liu X, Chen M, Zhang H, Wang PG. Cloning and characterization of GDP-perosamine synthetase (Per) from Escherichia coli O157:H7 and synthesis of GDP-perosamine in vitro. Biochem Biophys Res Commun. 2007 Nov 23;363(3):525–30.

91. Potter MD, Lo RY. Cloning and characterization of the galE locus of Pasteurella haemolytica A1. Infect Immun. 1996 Mar;64(3):855–60.

92. Lee H-C, Sohng J-K, Kim H-J, Nam D-H, Han J-M, Cho S-S, et al. Cloning and expression of the glucose-1-phosphate thymidylyltransferase gene (gerD) from Streptomyces sp. GERI-155. Mol Cells. 2004 Apr 30;17(2):274–80.

93. Karki S, Yoo H-G, Kwon S-Y, Suh J-W, Kwon H-J. Cloning and in vitro characterization of dTDP-6-deoxy-L-talose biosynthetic genes from Kitasatospora kifunensis featuring the dTDP-6-deoxy-L-lyxo-4-hexulose reductase that synthesizes dTDP-6-deoxy-L-talose. Carbohydr Res. 2010 Sep 3;345(13):1958–62.

94. Jennings MP, van der Ley P, Wilks KE, Maskell DJ, Poolman JT, Moxon ER. Cloning and molecular analysis of the galE gene of Neisseria meningitidis and its role in lipopolysaccharide biosynthesis. Mol Microbiol. 1993 Oct;10(2):361–9.

95. Griffin AM, Poelwijk ES, Morris VJ, Gasson MJ. Cloning of the aceF gene encoding the phosphomannose isomerase and GDP-mannose pyrophosphorylase activities involved in acetan biosynthesis in Acetobacter xylinum. FEMS Microbiol Lett. 1997 Sep 15;154(2):389–96.

96. Parajuli N, Lee D-S, Lee HC, Liou K, Sohng JK. Cloning, expression and characterization of glucose-1-phosphate thymidylyltransferase (strmlA) from Thermus caldophilus. Biotechnol Lett. 2004 Mar;26(5):437–42.

97. Ning B, Elbein AD. Cloning, expression and characterization of the pig liver GDP-mannose pyrophosphorylase. Evidence that GDP-mannose and GDP-Glc pyrophosphorylases are different proteins. Eur J Biochem. 2000 Dec;267(23):6866–74.

98. Sohng J-K, Kim H, Nam D-H, Lim D-O, Han J-M, Lee H-J, et al. Cloning, expression, and biological function of a dTDP-deoxyglucose epimerase (gerF) gene from Streptomyces sp. GERI-155. Biotechnol Lett. 2004 Feb;26(3):185–91.

99. Suryanti V, Nelson A, Berry A. Cloning, over-expression, purification, and

characterisation of N-acetylneuraminate synthase from Streptococcus agalactiae. Protein Expr Purif. 2003 Feb;27(2):346–56.

100. Thorson JS, Kelly TM, Liu HW. Cloning, sequencing, and overexpression in Escherichia coli of the alpha-D-glucose-1-phosphate cytidylyltransferase gene isolated from Yersinia pseudotuberculosis. J Bacteriol. 1994 Apr;176(7):1840–9.

101. Schollen E, Pardon E, Heykants L, Renard J, Doggett NA, Callen DF, et al. Comparative analysis of the phosphomannomutase genes PMM1, PMM2 and PMM2psi: the sequence variation in the processed pseudogene is a reflection of the mutations found in the functional gene. Hum Mol Genet. 1998 Feb;7(2):157–64.

102. Hung R-J, Chien H-S, Lin R-Z, Lin C-T, Vatsyayan J, Peng H-L, et al. Comparative analysis of two UDP-glucose dehydrogenases in Pseudomonas aeruginosa PAO1. J Biol Chem. 2007 Jun 15;282(24):17738–48.

103. Zhang P, Shao Z, Jin W, Duan D. Comparative characterization of two GDP-mannose dehydrogenase genes from Saccharina japonica (Laminariales, Phaeophyceae). BMC Plant Biol. 2016 Mar 8;16:62.

104. McCallum M, Shaw SD, Shaw GS, Creuzenet C. Complete 6-deoxy-D-altro-heptose biosynthesis pathway from Campylobacter jejuni: more complex than anticipated. J Biol Chem. 2012 Aug 24;287(35):29776–88.

105. Wei J, Goldberg MB, Burland V, Venkatesan MM, Deng W, Fournier G, et al. Complete genome sequence and comparative genomics of Shigella flexneri serotype 2a strain 2457T. Infect Immun. 2003 May;71(5):2775–86.

106. Chen Y-Y, Ko T-P, Lin C-H, Chen W-H, Wang AH-J. Conformational change upon product binding to Klebsiella pneumoniae UDP-glucose dehydrogenase: a possible inhibition mechanism for the key enzyme in polymyxin resistance. J Struct Biol. 2011 Sep;175(3):300–10.

107. Stern RJ, Lee T-Y, Lee T-J, Yan W, Scherman MS, Vissa VD, et al. Conversion of dTDP-4-keto-6-deoxyglucose to free dTDP-4-keto-rhamnose by the rmlC gene products of Escherichia coli and Mycobacterium tuberculosis. Microbiology. 1999 Mar;145 (Pt 3):663–71.

108. Rausch M, Deisinger JP, Ulm H, Müller A, Li W, Hardt P, et al. Coordination of capsule assembly and cell wall biosynthesis in Staphylococcus aureus. Nat Commun. 2019 Mar 29;10(1):1404.

109. Olivier NB, Imperiali B. Crystal structure and catalytic mechanism of PglD from Campylobacter jejuni. J Biol Chem. 2008 Oct 10;283(41):27937–46.

110. Riegler H, Herter T, Grishkovskaya I, Lude A, Ryngajllo M, Bolger ME, et al. Crystal structure and functional characterization of a glucosamine-6-phosphate.

Biochem J. 2012 Apr 15;443(2):427–37.

111. Vogan EM, Bellamacina C, He X, Liu H, Ringe D, Petsko GA. Crystal structure at 1.8 A resolution of CDP-D-glucose 4,6-dehydratase from Yersinia pseudotuberculosis. Biochemistry. 2004 Mar 23;43(11):3057–67.

112. Webb NA, Mulichak AM, Lam JS, Rocchetta HL, Garavito RM. Crystal structure of a tetrameric GDP-D-mannose 4,6-dehydratase from a bacterial. Protein Sci. 2004 Feb;13(2):529–39.

113. Mehra-Chaudhary R, Mick J, Beamer LJ. Crystal structure of Bacillus anthracis phosphoglucosamine mutase, an enzyme in the peptidoglycan biosynthetic pathway. J Bacteriol. 2011 Aug;193(16):4081–7.

114. Christendat D, Saridakis V, Dharamsi A, Bochkarev A, Pai EF, Arrowsmith CH, et al. Crystal structure of dTDP-4-keto-6-deoxy-D-hexulose 3,5-epimerase from Methanobacterium thermoautotrophicum complexed with dTDP. J Biol Chem. 2000 Aug 11;275(32):24608–12.

115. Gatzeva-Topalova PZ, May AP, Sousa MC. Crystal structure of Escherichia coli ArnA (PmrI) decarboxylase domain. A key enzyme for lipid A modification with 4-amino-4-deoxy-L-arabinose and polymyxin resistance. Biochemistry. 2004 Oct 26;43(42):13370–9.

116. Pampa KJ, Lokanath NK, Girish TU, Kunishima N, Rai VR. Crystal structure of product-bound complex of UDP-N-acetyl-d-mannosamine dehydrogenase from Pyrococcus horikoshii OT3. Biochem Biophys Res Commun. 2014 Oct 24;453(3):662–7.

117. Hung M-N, Rangarajan E, Munger C, Nadeau G, Sulea T, Matte A. Crystal structure of TDP-fucosamine acetyltransferase (WecD) from Escherichia coli, an enzyme required for enterobacterial common antigen synthesis. J Bacteriol. 2006 Aug;188(15):5606–17.

118. Ishiyama N, Creuzenet C, Lam JS, Berghuis AM. Crystal structure of WbpP, a genuine UDP-N-acetylglucosamine 4-epimerase from Pseudomonas aeruginosa: substrate specificity in udp-hexose 4-epimerases. J Biol Chem. 2004 May 21;279(21):22635–42.

119. Chen S-C, Huang C-H, Yang CS, Liu J-S, Kuan S-M, Chen Y. Crystal structures of the archaeal UDP-GlcNAc 2-epimerase from Methanocaldococcus jannaschii reveal a conformational change induced by UDP-GlcNAc. Proteins. 2014 Jul;82(7):1519–26.

120. Gross JW, Hegeman AD, Gerratana B, Frey PA. Dehydration is catalyzed by glutamate-136 and aspartic acid-135 active site residues in Escherichia coli dTDP-glucose 4,6-dehydratase. Biochemistry. 2001 Oct 23;40(42):12497–504.

121. Chen H, Thomas MG, Hubbard BK, Losey HC, Walsh CT, Burkart MD. Deoxysugars in glycopeptide antibiotics: enzymatic synthesis of TDP-L-epivancosamine in chloroeremomycin biosynthesis. Proc Natl Acad Sci U S A. 2000 Oct 24;97(22):11942–7.

122. Ma Y, Mills JA, Belisle JT, Vissa V, Howell M, Bowlin K, et al. Determination of the pathway for rhamnose biosynthesis in mycobacteria: cloning, sequencing and expression of the Mycobacterium tuberculosis gene encoding alpha-D-glucose-1-phosphate thymidylyltransferase. Microbiology. 1997 Mar;143 (Pt 3):937–45.

123. Brokate-Llanos AM, Monje JM, Murdoch PDS, Muñoz MJ. Developmental defects in a Caenorhabditis elegans model for type III galactosemia. Genetics. 2014 Dec;198(4):1559–69.

124. Fruscione F, Sturla L, Duncan G, Van Etten JL, Valbuzzi P, De Flora A, et al. Differential role of NADP+ and NADPH in the activity and structure of GDP-D-mannose 4,6-dehydratase from two chlorella viruses. J Biol Chem. 2008 Jan 4;283(1):184–93.

125. Barber C, Rösti J, Rawat A, Findlay K, Roberts K, Seifert GJ. Distinct properties of the five UDP-D-glucose/UDP-D-galactose 4-epimerase isoforms of Arabidopsis thaliana. J Biol Chem. 2006 Jun 23;281(25):17276–85.

126. Wang L, Huang H, Nguyen HH, Allen KN, Mariano PS, Dunaway-Mariano D. Divergence of biochemical function in the HAD superfamily: Biochemistry. 2010 Feb 16;49(6):1072–81.

127. Reboul R, Geserick C, Pabst M, Frey B, Wittmann D, Lütz-Meindl U, et al. Down-regulation of UDP-glucuronic acid biosynthesis leads to swollen plant cell walls and severe developmental defects associated with changes in pectic polysaccharides. J Biol Chem. 2011 Nov 18;286(46):39982–92.

128. Miyafusa T, Caaveiro JMM, Tanaka Y, Tsumoto K. Dynamic elements govern the catalytic activity of CapE, a capsular polysaccharide-synthesizing enzyme from Staphylococcus aureus. FEBS Lett. 2013 Nov 29;587(23):3824–30.

129. Kang J, Xu L, Yang S, Yu W, Liu S, Xin Y, et al. Effect of phosphoglucosamine mutase on biofilm formation and antimicrobial susceptibilities in M. smegmatis glmM gene knockdown strain. PLoS One. 2013;8(4):e61589.

130. Butty FD, Aucoin M, Morrison L, Ho N, Shaw G, Creuzenet C. Elucidating the formation of 6-deoxyheptose: biochemical characterization of the. Biochemistry. 2009 Aug 18;48(32):7764–75.

131. Schoenhofen IC, McNally DJ, Brisson J-R, Logan SM. Elucidation of the CMP-pseudaminic acid pathway in Helicobacter pylori: synthesis from UDP-N-acetylglucosamine by a single enzymatic reaction. Glycobiology. 2006 Sep;16(9):8C-14C.

132. Gu X, Wages CJ, Davis KE, Guyett PJ, Bar-Peled M. Enzymatic characterization and comparison of various poaceae UDP-GlcA 4-epimerase isoforms. J Biochem. 2009 Oct;146(4):527–34.

133. Kawamura T, Ishimoto N, Ito E. Enzymatic synthesis of uridine diphosphate N-acetyl-D-mannosaminuronic acid. J Biol Chem. 1979 Sep 10;254(17):8457–65.

134. Kaundinya CR, Savithri HS, Rao KK, Balaji PV. EpsM from Bacillus subtilis 168 has UDP-2,4,6-trideoxy-2-acetamido-4-amino glucose acetyltransferase activity in vitro. Biochem Biophys Res Commun. 2018 Nov 10;505(4):1057–62.

135. Kaundinya CR, Savithri HS, Rao KK, Balaji PV. EpsN from Bacillus subtilis 168 has UDP-2,6-dideoxy 2-acetamido 4-keto glucose aminotransferase activity in vitro. Glycobiology. 2018 Oct 1;28(10):802–12.

136. Albermann C, Piepersberg W. Expression and identification of the RfbE protein from Vibrio cholerae O1 and its use for the enzymatic synthesis of GDP-D-perosamine. Glycobiology. 2001 Aug;11(8):655–61.

137. Viswanathan K, Tomiya N, Park J, Singh S, Lee YC, Palter K, et al. Expression of a functional Drosophila melanogaster CMP-sialic acid synthetase. Differential localization of the Drosophila and human enzymes. J Biol Chem. 2006 Jun 9;281(23):15929–40.

138. Kim K, Lawrence SM, Park J, Pitts L, Vann WF, Betenbaugh MJ, et al. Expression of a functional Drosophila melanogaster N-acetylneuraminic acid (Neu5Ac) phosphate synthase gene: evidence for endogenous sialic acid biosynthetic ability in insects. Glycobiology. 2002 Feb;12(2):73–83.

139. Swartley JS, Ahn JH, Liu LJ, Kahler CM, Stephens DS. Expression of sialic acid and polysialic acid in serogroup B Neisseria meningitidis: divergent transcription of biosynthesis and transport operons through a common promoter region. J Bacteriol. 1996 Jul;178(14):4052–9.

140. Weisser P, Krämer R, Sprenger GA. Expression of the Escherichia coli pmi gene, encoding phosphomannose-isomerase in Zymomonas mobilis, leads to utilization of mannose as a novel growth substrate, which can be used as a selective marker. Appl Environ Microbiol. 1996 Nov;62(11):4155–61.

141. Zhang W, Jones VC, Scherman MS, Mahapatra S, Crick D, Bhamidi S, et al. Expression, essentiality, and a microtiter plate assay for mycobacterial GlmU, the bifunctional glucosamine-1-phosphate acetyltransferase and. Int J Biochem Cell Biol. 2008;40(11):2560–71.

142. Sturla L, Bisso A, Zanardi D, Benatti U, De Flora A, Tonetti M. Expression, purification and characterization of GDP-D-mannose 4,6-dehydratase from Escherichia coli. FEBS Lett. 1997 Jul 21;412(1):126–30.

143. Elling L, Ritter JE, Verseck S. Expression, purification and characterization of recombinant phosphomannomutase and. Glycobiology. 1996 Sep;6(6):591–7.

144. Lai X, Wu J, Chen S, Zhang X, Wang H. Expression, purification, and characterization of a functionally active Mycobacterium tuberculosis UDP-glucose pyrophosphorylase. Protein Expr Purif. 2008 Sep;61(1):50–6.

145. Allen JG, Mujacic M, Frohn MJ, Pickrell AJ, Kodama P, Bagal D, et al. Facile Modulation of Antibody Fucosylation with Small Molecule Fucostatin Inhibitors and Cocrystal Structure with GDP-Mannose 4,6-Dehydratase. ACS Chem Biol. 2016 Oct 21;11(10):2734–43.

146. Muñoz R, López R, de Frutos M, García E. First molecular characterization of a uridine diphosphate galacturonate 4-epimerase: an enzyme required for capsular biosynthesis in Streptococcus pneumoniae type 1. Mol Microbiol. 1999 Jan;31(2):703–13.

147. Ma Y, Pan F, McNeil M. Formation of dTDP-rhamnose is essential for growth of mycobacteria. J Bacteriol. 2002 Jun;184(12):3392–5.

148. Yin S, Liu M, Kong J-Q. Functional analyses of OcRhS1 and OcUER1 involved in UDP-L-rhamnose biosynthesis in Ornithogalum caudatum. Plant Physiol Biochem. 2016 Dec;109:536–48.

149. Oka T, Nemoto T, Jigami Y. Functional analysis of Arabidopsis thaliana RHM2/MUM4, a multidomain protein involved in UDP-D-glucose to UDP-L-rhamnose conversion. J Biol Chem. 2007 Feb 23;282(8):5389–403.

150. Sousa SA, Moreira LM, Leitão JH. Functional analysis of the Burkholderia cenocepacia J2315 BceAJ protein with phosphomannose isomerase and GDP-D-mannose pyrophosphorylase activities. Appl Microbiol Biotechnol. 2008 Oct;80(6):1015–22.

151. Schoenhofen IC, McNally DJ, Vinogradov E, Whitfield D, Young NM, Dick S, et al. Functional characterization of dehydratase/aminotransferase pairs from Helicobacter and Campylobacter: enzymes distinguishing the pseudaminic acid and bacillosamine biosynthetic pathways. J Biol Chem. 2006 Jan 13;281(2):723–32.

152. Bengoechea JA, Pinta E, Salminen T, Oertelt C, Holst O, Radziejewska-Lebrecht J, et al. Functional characterization of Gne (UDP-N-acetylglucosamine-4-epimerase), Wzz (chain length determinant), and Wzy (O-antigen polymerase) of Yersinia enterocolitica serotype O:8. J Bacteriol. 2002 Aug;184(15):4277–87.

153. Thuy TTT, Lee HC, Kim C-G, Heide L, Sohng JK. Functional characterizations of novWUS involved in novobiocin biosynthesis from Streptomyces spheroides. Arch Biochem Biophys. 2005 Apr 1;436(1):161–7.

154. Bar-Peled M, Griffith CL, Doering TL. Functional cloning and characterization of a UDP- glucuronic acid decarboxylase: the pathogenic fungus Cryptococcus neoformans elucidates UDP-xylose synthesis. Proc Natl Acad Sci U S A. 2001 Oct 9;98(21):12003–8.

155. Mio T, Yamada-Okabe T, Arisawa M, Yamada-Okabe H. Functional cloning and mutational analysis of the human cDNA for phosphoacetylglucosamine mutase: identification of the amino acid residues essential for the catalysis. Biochim Biophys Acta. 2000 Jul 24;1492(2–3):369–76.

156. Mäki M, Järvinen N, Räbinä J, Roos C, Maaheimo H, Renkonen R. Functional expression of Pseudomonas aeruginosa GDP-4-keto-6-deoxy-D-mannose reductase which synthesizes GDP-rhamnose. Eur J Biochem. 2002 Jan;269(2):593–601.

157. Wang Z, Wang Y, Hong X, Hu D, Liu C, Yang J, et al. Functional inactivation of UDP-N-acetylglucosamine pyrophosphorylase 1 (UAP1) induces early leaf senescence and defence responses in rice. J Exp Bot. 2015 Feb;66(3):973–87.

158. Graack HR, Cinque U, Kress H. Functional regulation of glutamine:fructose-6-phosphate aminotransferase 1 (GFAT1) of Drosophila melanogaster in a UDP-N-acetylglucosamine and cAMP-dependent manner. Biochem J. 2001 Dec 1;360(Pt 2):401–12.

159. van der Beek SL, Le Breton Y, Ferenbach AT, Chapman RN, van Aalten DMF, Navratilova I, et al. GacA is essential for Group A Streptococcus and defines a new class of monomeric dTDP-4-dehydrorhamnose reductases (RmlD). Mol Microbiol. 2015 Dec;98(5):946–62.

160. Nassau PM, Martin SL, Brown RE, Weston A, Monsey D, McNeil MR, et al. Galactofuranose biosynthesis in Escherichia coli K-12: identification and cloning of. J Bacteriol. 1996 Feb;178(4):1047–52.

161. Cook PD, Holden HM. GDP-4-keto-6-deoxy-D-mannose 3-dehydratase, accommodating a sugar substrate in the active site. J Biol Chem. 2008 Feb 15;283(7):4295–303.

162. Qin C, Qian W, Wang W, Wu Y, Yu C, Jiang X, et al. GDP-mannose pyrophosphorylase is a genetic determinant of ammonium sensitivity in Arabidopsis thaliana. Proc Natl Acad Sci U S A. 2008 Nov 25;105(47):18308–13.

163. Jiang H, Ouyang H, Zhou H, Jin C. GDP-mannose pyrophosphorylase is essential for cell wall integrity, morphogenesis and viability of Aspergillus fumigatus. Microbiology. 2008 Sep;154(Pt 9):2730–9.

164. Denton H, Fyffe S, Smith TK. GDP-mannose pyrophosphorylase is essential in the bloodstream form of Trypanosoma brucei. Biochem J. 2010 Jan 15;425(3):603–14.

165. Zhang Q, Hrmova M, Shirley NJ, Lahnstein J, Fincher GB. Gene expression patterns and catalytic properties of UDP-D-glucose 4-epimerases from barley (Hordeum vulgare L.). Biochem J. 2006 Feb 15;394(Pt 1):115–24.

166. Nguyen LC, Yamamoto M, Ohnishi-Kameyama M, Andi S, Taguchi F, Iwaki M, et al. Genetic analysis of genes involved in synthesis of modified. Mol Genet Genomics. 2009 Dec;282(6):595–605.

167. Kim S-H, Ahn S-H, Lee J-H, Lee E-M, Kim N-H, Park K-J, et al. Genetic analysis of phosphomannomutase/phosphoglucomutase from Vibrio furnissii and characterization of its role in virulence. Arch Microbiol. 2003 Oct;180(4):240–50.

168. Marolda CL, Valvano MA. Genetic analysis of the dTDP-rhamnose biosynthesis region of the Escherichia coli VW187 (O7:K1) rfb gene cluster: identification of functional homologs of rfbB and rfbA in the rff cluster and correct location of the rffE gene. J Bacteriol. 1995 Oct;177(19):5539–46.

169. Liu B, Chen M, Perepelov AV, Liu J, Ovchinnikova OG, Zhou D, et al. Genetic analysis of the O-antigen of Providencia alcalifaciens O30 and biochemical characterization of a formyltransferase involved in the synthesis of a Qui4N derivative. Glycobiology. 2012 Sep;22(9):1236–44.

170. James DBA, Yother J. Genetic and biochemical characterizations of enzymes involved in Streptococcus pneumoniae serotype 2 capsule synthesis demonstrate that Cps2T (WchF) catalyzes the committed step by addition of β1-4 rhamnose, the second sugar residue in the repeat unit. J Bacteriol. 2012 Dec;194(23):6479–89.

171. Fang W, Du T, Raimi OG, Hurtado-Guerrero R, Urbaniak MD, Ibrahim AFM, et al. Genetic and structural validation of Aspergillus fumigatus UDP-N-acetylglucosamine pyrophosphorylase as an antifungal target. Mol Microbiol. 2013 Aug;89(3):479–93.

172. Köplin R, Arnold W, Hötte B, Simon R, Wang G, Pühler A. Genetics of xanthan production in Xanthomonas campestris: the xanA and xanB genes are involved in UDP-glucose and GDP-mannose biosynthesis. J Bacteriol. 1992 Jan;174(1):191–9.

173. Jin Q, Yuan Z, Xu J, Wang Y, Shen Y, Lu W, et al. Genome sequence of Shigella flexneri 2a: insights into pathogenicity through comparison with genomes of Escherichia coli K12 and O157. Nucleic Acids Res. 2002 Oct 15;30(20):4432–41.

174. Piacente F, Marin M, Molinaro A, De Castro C, Seltzer V, Salis A, et al. Giant DNA virus mimivirus encodes pathway for biosynthesis of unusual sugar. J Biol Chem. 2012 Jan 27;287(5):3009–18.

175. Piacente F, De Castro C, Jeudy S, Molinaro A, Salis A, Damonte G, et al. Giant virus Megavirus chilensis encodes the biosynthetic pathway for uncommon

acetamido sugars. J Biol Chem. 2014 Aug 29;289(35):24428–39.

176. Plata G, Fuhrer T, Hsiao T-L, Sauer U, Vitkup D. Global probabilistic annotation of metabolic networks enables enzyme discovery. Nat Chem Biol. 2012 Oct;8(10):848–54.

177. Badet B, Vermoote P, Haumont PY, Lederer F, LeGoffic F. Glucosamine synthetase from Escherichia coli: purification, properties, and glutamine-utilizing site location. Biochemistry. 1987 Apr 7;26(7):1940–8.

178. Suzuki N, Nakano Y, Yoshida Y, Nezu T, Terada Y, Yamashita Y, et al. Guanosine diphosphate-4-keto-6-deoxy-d-mannose reductase in the pathway for the synthesis of GDP-6-deoxy-d-talose in Actinobacillus actinomycetemcomitans. Eur J Biochem. 2002 Dec;269(23):5963–71.

179. Kaminski L, Eichler J. Haloferax volcanii N-glycosylation: delineating the pathway of dTDP-rhamnose biosynthesis. PLoS One. 2014;9(5):e97441.

180. Allard STM, Cleland WW, Holden HM. High resolution X-ray structure of dTDP-glucose 4,6-dehydratase from Streptomyces venezuelae. J Biol Chem. 2004 Jan 16;279(3):2211–20.

181. Koropatkin NM, Liu H-W, Holden HM. High resolution x-ray structure of tyvelose epimerase from Salmonella typhi. J Biol Chem. 2003 Jun 6;278(23):20874–81.

182. Dong C, Major LL, Allen A, Blankenfeldt W, Maskell D, Naismith JH. High-resolution structures of RmlC from Streptococcus suis in complex with substrate analogs locate the active site of this class of enzyme. Structure. 2003 Jun;11(6):715–23.

183. Graninger M, Kneidinger B, Bruno K, Scheberl A, Messner P. Homologs of the Rml enzymes from Salmonella enterica are responsible for dTDP-beta-L-rhamnose biosynthesis in the gram-positive thermophile Aneurinibacillus thermoaerophilus DSM 10155. Appl Environ Microbiol. 2002 Aug;68(8):3708–15.

184. Mijakovic I, Petranovic D, Deutscher J. How tyrosine phosphorylation affects the UDP-glucose dehydrogenase activity of Bacillus subtilis YwqF. J Mol Microbiol Biotechnol. 2004;8(1):19–25.

185. Thoden JB, Wohlers TM, Fridovich-Keil JL, Holden HM. Human UDP-galactose 4-epimerase. Accommodation of UDP-N-acetylglucosamine within the active site. J Biol Chem. 2001 May 4;276(18):15131–6.

186. Schaper W, Bentrop J, Ustinova J, Blume L, Kats E, Tiralongo J, et al. Identification and biochemical characterization of two functional CMP-sialic acid synthetases in Danio rerio. J Biol Chem. 2012 Apr 13;287(16):13239–48.

187. Westman EL, McNally DJ, Rejzek M, Miller WL, Kannathasan VS, Preston A, et al. Identification and biochemical characterization of two novel. Biochem J. 2007 Jul 1;405(1):123–30.

188. Yang T, Echols M, Martin A, Bar-Peled M. Identification and characterization of a strict and a promiscuous. Biochem J. 2010 Sep 1;430(2):275–84.

189. Usadel B, Schlüter U, Mølhøj M, Gipmans M, Verma R, Kossmann J, et al. Identification and characterization of a UDP-D-glucuronate 4-epimerase in Arabidopsis. FEBS Lett. 2004 Jul 2;569(1–3):327–31.

190. Wu B, Zhang Y, Wang PG. Identification and characterization of GDP-d-mannose 4,6-dehydratase and. Biochem Biophys Res Commun. 2001 Jul 13;285(2):364–71.

191. Chou WK, Dick S, Wakarchuk WW, Tanner ME. Identification and characterization of NeuB3 from Campylobacter jejuni as a pseudaminic acid synthase. J Biol Chem. 2005 Oct 28;280(43):35922–8.

192. Wills EA, Roberts IS, Del Poeta M, Rivera J, Casadevall A, Cox GM, et al. Identification and characterization of the Cryptococcus neoformans phosphomannose isomerase-encoding gene, MAN1, and its impact on pathogenicity. Mol Microbiol. 2001 May;40(3):610–20.

193. Kawano Y, Sekine M, Ihara M. Identification and characterization of UDP-glucose pyrophosphorylase in cyanobacteria Anabaena sp. PCC 7120. J Biosci Bioeng. 2014 May;117(5):531–8.

194. Murkin AS, Chou WK, Wakarchuk WW, Tanner ME. Identification and mechanism of a bacterial hydrolyzing UDP-N-acetylglucosamine. Biochemistry. 2004 Nov 9;43(44):14290–8.

195. Dadashipour M, Iwamoto M, Hossain MM, Akutsu J-I, Zhang Z, Kawarabayasi Y. Identification of a Direct Biosynthetic Pathway for UDP-N-Acetylgalactosamine from Glucosamine-6-Phosphate in Thermophilic Crenarchaeon Sulfolobus tokodaii. J Bacteriol. 2018 May 15;200(10).

196. Feng L, Shou Q, Butcher RA. Identification of a dTDP-rhamnose biosynthetic pathway that oscillates with the molting cycle in Caenorhabditis elegans. Biochem J. 2016 Jun 1;473(11):1507–21.

197. Sacchetti S, Bartolucci S, Rossi M, Cannio R. Identification of a GDP-mannose pyrophosphorylase gene from Sulfolobus solfataricus. Gene. 2004 May 12;332:149–57.

198. Qi X-Q, Sun Q-L, Bai L-P, Shan J-J, Zhang Y, Zhang R, et al. Identification of alpha-D-glucose-1-phosphate cytidylyltransferase involved in Ebosin biosynthesis of Streptomyces sp. 139. Appl Microbiol Biotechnol. 2009 May;83(2):361–8.

199. Zhang Z, Tsujimura M, Akutsu J, Sasaki M, Tajima H, Kawarabayasi Y. Identification of an extremely thermostable enzyme with dual sugar-1-phosphate nucleotidylyltransferase activities from an acidothermophilic archaeon, Sulfolobus tokodaii strain 7. J Biol Chem. 2005 Mar 11;280(10):9698–705.

200. Parakkottil Chothi M, Duncan GA, Armirotti A, Abergel C, Gurnon JR, Van Etten JL, et al. Identification of an L-rhamnose synthetic pathway in two nucleocytoplasmic large DNA viruses. J Virol. 2010 Sep;84(17):8829–38.

201. Li S, Kang J, Yu W, Zhou Y, Zhang W, Xin Y, et al. Identification of M. tuberculosis Rv3441c and M. smegmatis MSMEG_1556 and essentiality of M. smegmatis MSMEG_1556. PLoS One. 2012;7(8):e42769.

202. Nishimoto M, Kitaoka M. Identification of N-acetylhexosamine 1-kinase in the complete lacto-N-biose I/galacto-N-biose metabolic pathway in Bifidobacterium longum. Appl Environ Microbiol. 2007 Oct;73(20):6444–9.

203. Albermann C, Beuttler H. Identification of the GDP-N-acetyl-d-perosamine producing enzymes from Escherichia coli O157:H7. FEBS Lett. 2008 Feb 20;582(4):479–84.

204. Godfroid F, Taminiau B, Danese I, Denoel P, Tibor A, Weynants V, et al. Identification of the perosamine synthetase gene of Brucella melitensis 16M and involvement of lipopolysaccharide O side chain in Brucella survival in mice and in macrophages. Infect Immun. 1998 Nov;66(11):5485–93.

205. Videira PA, Cortes LL, Fialho AM, Sá-Correia I. Identification of the pgmG gene, encoding a bifunctional protein with phosphoglucomutase and phosphomannomutase activities, in the gellan gum-producing strain Sphingomonas paucimobilis ATCC 31461. Appl Environ Microbiol. 2000 May;66(5):2252–8.

206. Tavares IM, Jolly L, Pompeo F, Leitão JH, Fialho AM, Sá-Correia I, et al. Identification of the Pseudomonas aeruginosa glmM gene, encoding phosphoglucosamine mutase. J Bacteriol. 2000 Aug;182(16):4453–7.

207. Shimazu K, Takahashi Y, Uchikawa Y, Shimazu Y, Yajima A, Takashima E, et al. Identification of the Streptococcus gordonii glmM gene encoding phosphoglucosamine mutase and its role in bacterial cell morphology, biofilm formation, and sensitivity to antibiotics. FEMS Immunol Med Microbiol. 2008 Jul;53(2):166–77.

208. Dong S, Chesnokova ON, Turnbough CLJ, Pritchard DG. Identification of the UDP-N-acetylglucosamine 4-epimerase involved in exosporium protein glycosylation in Bacillus anthracis. J Bacteriol. 2009 Nov;191(22):7094–101.

209. Park NY, Lee JH, Kim MW, Jeong HG, Lee BC, Kim TS, et al. Identification of the Vibrio vulnificus wbpP gene and evaluation of its role in virulence. Infect

Immun. 2006 Jan;74(1):721–8.

210. Kneidinger B, Graninger M, Adam G, Puchberger M, Kosma P, Zayni S, et al. Identification of two GDP-6-deoxy-D-lyxo-4-hexulose reductases synthesizing. J Biol Chem. 2001 Feb 23;276(8):5577–83.

211. Mariño K, Güther MLS, Wernimont AK, Amani M, Hui R, Ferguson MAJ. Identification, subcellular localization, biochemical properties, and high-resolution crystal structure of Trypanosoma brucei UDP-glucose pyrophosphorylase. Glycobiology. 2010 Dec;20(12):1619–30.

212. Li T, Simonds L, Kovrigin EL, Noel KD. In vitro biosynthesis and chemical identification of UDP-N-acetyl-d-quinovosamine (UDP-d-QuiNAc). J Biol Chem. 2014 Jun 27;289(26):18110–20.

213. Kaundinya CR, Savithri HS, Krishnamurthy Rao K, Balaji PV. In vitro characterization of N-terminal truncated EpsC from Bacillus subtilis 168, a. Arch Biochem Biophys. 2018 Nov 1;657:78–88.

214. Hong L, Zhao Z, Melançon CE 3rd, Zhang H, Liu H. In vitro characterization of the enzymes involved in TDP-D-forosamine biosynthesis in the spinosyn pathway of Saccharopolyspora spinosa. J Am Chem Soc. 2008 Apr 9;130(14):4954–67.

215. Lindqvist L, Schweda KH, Reeves PR, Lindberg AA. In vitro synthesis of CDP-d-abequose using Salmonella enzymes of cloned rfb genes. Production of CDP-6-deoxy-D-xylo-4-hexulose, CDP-3,6-dideoxy-D-xylo-4-hexulose and. Eur J Biochem. 1994 Nov 1;225(3):863–72.

216. Liu F, Lee HJ, Strynadka NCJ, Tanner ME. Inhibition of Neisseria meningitidis sialic acid synthase by a tetrahedral intermediate analogue. Biochemistry. 2009 Oct 6;48(39):9194–201.

217. Green OM, McKenzie AR, Shapiro AB, Otterbein L, Ni H, Patten A, et al. Inhibitors of acetyltransferase domain of. Bioorg Med Chem Lett. 2012 Feb 15;22(4):1510–9.

218. Chen H, Zhao Z, Hallis TM, Guo Z, Liu Hw H. Insights into the Branched-Chain Formation of Mycarose: Methylation Catalyzed by an (S)-Adenosylmethionine-Dependent Methyltransferase We are grateful to Dr. Eugene Seno and the Lilly Research Laboratories for their generous gift of the plasmid pHJL311 and to the National Institutes of Health for grants (GM 35 906 and 54 346). H.-w.L. also thanks the National Institute of General Medical Sciences for a MERIT Award. T.M.H. was a trainee of the National Institute of General Medical Sciences (Biotechnology Training Grant: 2 T32 GM08347). Angew Chem Int Ed Engl. 2001 Feb 2;40(3):607–10.

219. Hofmeister DL, Thoden JB, Holden HM. Investigation of a sugar N-formyltransferase from the plant pathogen Pantoea ananatis. Protein Sci. 2019

Apr;28(4):707–16.

220. Teplyakov A, Obmolova G, Badet-Denisot MA, Badet B, Polikarpov I. Involvement of the C terminus in intramolecular nitrogen channeling in glucosamine. Structure. 1998 Aug 15;6(8):1047–55.

221. Smith RJ, Milewski S, Brown AJ, Gooday GW. Isolation and characterization of the GFA1 gene encoding the glutamine:fructose-6-phosphate amidotransferase of Candida albicans. J Bacteriol. 1996 Apr;178(8):2320–7.

222. Zuccotti S, Zanardi D, Rosano C, Sturla L, Tonetti M, Bolognesi M. Kinetic and crystallographic analyses support a sequential-ordered bi bi catalytic mechanism for Escherichia coli glucose-1-phosphate thymidylyltransferase. J Mol Biol. 2001 Nov 2;313(4):831–43.

223. Koropatkin NM, Cleland WW, Holden HM. Kinetic and structural analysis of alpha-D-Glucose-1-phosphate cytidylyltransferase from Salmonella typhi. J Biol Chem. 2005 Mar 18;280(11):10774–80.

224. Bravo IG, Barrallo S, Ferrero MA, Rodríguez-Aparicio LB, Martínez-Blanco H, Reglero A. Kinetic properties of the acylneuraminate cytidylyltransferase from Pasteurella haemolytica A2. Biochem J. 2001 Sep 15;358(Pt 3):585–98.

225. Gassner GT, Johnson DA, Liu HW, Ballou DP. Kinetics of the reductive half-reaction of the iron-sulfur flavoenzyme. Biochemistry. 1996 Jun 18;35(24):7752–61.

226. Zhou D, Stephens DS, Gibson BW, Engstrom JJ, McAllister CF, Lee FK, et al. Lipooligosaccharide biosynthesis in pathogenic Neisseria. Cloning, identification, and characterization of the phosphoglucomutase gene. J Biol Chem. 1994 Apr 15;269(15):11162–9.

227. Yang Y-H, Song E, Park S-H, Kim J-N, Lee K, Kim E, et al. Loss of phosphomannomutase activity enhances actinorhodin production in Streptomyces coelicolor. Appl Microbiol Biotechnol. 2010 May;86(5):1485–92.

228. Walsh RMJ, Polizzi SJ, Kadirvelraj R, Howard WW, Wood ZA. Man o' war mutation in UDP-α-D-xylose synthase favors the abortive catalytic cycle and uncovers a latent potential for hexamer formation. Biochemistry. 2015 Jan 27;54(3):807–19.

229. Weigel TM, Liu LD, Liu HW. Mechanistic studies of the biosynthesis of 3,6-dideoxyhexoses in Yersinia pseudotuberculosis: purification and characterization of. Biochemistry. 1992 Feb 25;31(7):2129–39.

230. Hallis TM, Lei Y, Que NL, Liu H. Mechanistic studies of the biosynthesis of paratose: purification and characterization of CDP-paratose synthase. Biochemistry. 1998 Apr 7;37(14):4935–45.

231. Lei Y, Ploux O, Liu HW. Mechanistic studies on CDP-6-deoxy-L-threo-D-glycero-4-hexulose 3-dehydrase identification of His-220 as the active-site base by chemical modification and site-directed mutagenesis. Biochemistry. 1995 Apr 11;34(14):4643–54.

232. Pageni BB, Oh T-J, Lee HC, Sohng JK. Metabolic engineering of noviose: heterologous expression of novWUS and generation of a new hybrid antibiotic, noviosylated 10-deoxymethynolide/narbonolide, from Streptomyces venezuelae YJ003-OTBP1. Biotechnol Lett. 2008 Sep;30(9):1609–15.

233. Lee FK, Stephens DS, Gibson BW, Engstrom JJ, Zhou D, Apicella MA. Microheterogeneity of Neisseria lipooligosaccharide: analysis of a UDP-glucose. Infect Immun. 1995 Jul;63(7):2508–15.

234. Qian W, Yu C, Qin H, Liu X, Zhang A, Johansen IE, et al. Molecular and functional analysis of phosphomannomutase (PMM) from higher plants and genetic evidence for the involvement of PMM in ascorbic acid biosynthesis in Arabidopsis and Nicotiana benthamiana. Plant J. 2007 Feb;49(3):399–413.

235. Woodford CR, Thoden JB, Holden HM. Molecular architecture of an N-formyltransferase from Salmonella enterica O60. J Struct Biol. 2017 Dec;200(3):267–78.

236. Burgie ES, Holden HM. Molecular architecture of DesI: a key enzyme in the biosynthesis of desosamine. Biochemistry. 2007 Aug 7;46(31):8999–9006.

237. Burgie ES, Thoden JB, Holden HM. Molecular architecture of DesV from Streptomyces venezuelae: a PLP-dependent transaminase involved in the biosynthesis of the unusual sugar desosamine. Protein Sci. 2007 May;16(5):887–96.

238. García García MI, Lau K, von Itzstein M, García Carmona F, Sánchez Ferrer Á. Molecular characterization of a new N-acetylneuraminate synthase (NeuB1) from Idiomarina loihiensis. Glycobiology. 2015 Jan;25(1):115–23.

239. Yeom S-J, Kim Y-S, Lim Y-R, Jeong K-W, Lee J-Y, Kim Y, et al. Molecular characterization of a novel thermostable mannose-6-phosphate isomerase from Thermus thermophilus. Biochimie. 2011 Oct;93(10):1659–67.

240. Crater DL, Dougherty BA, van de Rijn I. Molecular characterization of hasC from an operon required for hyaluronic acid synthesis in group A streptococci. Demonstration of UDP-glucose pyrophosphorylase activity. J Biol Chem. 1995 Dec 1;270(48):28676–80.

241. Ma Z, Fan H, Lu C. Molecular cloning and analysis of the UDP-Glucose Pyrophosphorylase in Streptococcus equi subsp. zooepidemicus. Mol Biol Rep. 2011 Apr;38(4):2751–60.

242. Spicer AP, Kaback LA, Smith TJ, Seldin MF. Molecular cloning and characterization of the human and mouse UDP-glucose dehydrogenase genes. J Biol Chem. 1998 Sep 25;273(39):25117–24.

243. Nakata D, Münster AK, Gerardy-Schahn R, Aoki N, Matsuda T, Kitajima K. Molecular cloning of a unique CMP-sialic acid synthetase that effectively utilizes both deaminoneuraminic acid (KDN) and N-acetylneuraminic acid (Neu5Ac) as substrates. Glycobiology. 2001 Aug;11(8):685–92.

244. Sullivan FX, Kumar R, Kriz R, Stahl M, Xu GY, Rouse J, et al. Molecular cloning of human GDP-mannose 4,6-dehydratase and reconstitution of. J Biol Chem. 1998 Apr 3;273(14):8193–202.

245. Jensen SO, Reeves PR. Molecular evolution of the GDP-mannose pathway genes (manB and manC) in Salmonella enterica. Microbiology. 2001 Mar;147(Pt 3):599–610.

246. Thoden JB, Holden HM. Molecular structure of WlbB, a bacterial N-acetyltransferase involved in the biosynthesis of 2,3-diacetamido-2,3-dideoxy-D-mannuronic acid . Biochemistry. 2010 Jun 8;49(22):4644–53.

247. Linton D, Karlyshev AV, Hitchen PG, Morris HR, Dell A, Gregson NA, et al. Multiple N-acetyl neuraminic acid synthetase (neuB) genes in Campylobacter jejuni: identification and characterization of the gene involved in sialylation of lipo-oligosaccharide. Mol Microbiol. 2000 Mar;35(5):1120–34.

248. Humphreys GB, Jud MC, Monroe KM, Kimball SS, Higley M, Shipley D, et al. Mummy, A UDP-N-acetylglucosamine pyrophosphorylase, modulates DPP signaling in the embryonic epidermis of Drosophila. Dev Biol. 2013 Sep 15;381(2):434–45.

249. Yurist-Doutsch S, Magidovich H, Ventura VV, Hitchen PG, Dell A, Eichler J. N-glycosylation in Archaea: on the coordinated actions of Haloferax volcanii AglF and AglM. Mol Microbiol. 2010 Feb;75(4):1047–58.

250. van Karnebeek CDM, Bonafé L, Wen X-Y, Tarailo-Graovac M, Balzano S, Royer-Bertrand B, et al. NANS-mediated synthesis of sialic acid is required for brain and skeletal development. Nat Genet. 2016 Jul;48(7):777–84.

251. Woodford CR, Thoden JB, Holden HM. New role for the ankyrin repeat revealed by a study of the N-formyltransferase from Providencia alcalifaciens. Biochemistry. 2015 Jan 27;54(3):631–8.

252. Kowal P, Wang PG. New UDP-GlcNAc C4 epimerase involved in the biosynthesis of. Biochemistry. 2002 Dec 24;41(51):15410–4.

253. Babaoglu K, Page MA, Jones VC, McNeil MR, Dong C, Naismith JH, et al. Novel inhibitors of an emerging target in Mycobacterium tuberculosis; substituted

thiazolidinones as inhibitors of dTDP-rhamnose synthesis. Bioorg Med Chem Lett. 2003 Oct 6;13(19):3227–30.

254. Kereszt A, Kiss E, Reuhs BL, Carlson RW, Kondorosi A, Putnoky P. Novel rkp gene clusters of Sinorhizobium meliloti involved in capsular polysaccharide production and invasion of the symbiotic nodule: the rkpK gene encodes a UDP-glucose dehydrogenase. J Bacteriol. 1998 Oct;180(20):5426–31.

255. Munster A-K, Weinhold B, Gotza B, Muhlenhoff M, Frosch M, Gerardy-Schahn R. Nuclear localization signal of murine CMP-Neu5Ac synthetase includes residues required for both nuclear targeting and enzymatic activity. J Biol Chem. 2002 May 31;277(22):19688–96.

256. Miles JS, Guest JR. Nucleotide sequence and transcriptional start point of the phosphomannose isomerase gene (manA) of Escherichia coli. Gene. 1984 Dec;32(1–2):41–8.

257. Hayashi H, Araki Y, Ito E. Occurrence of glucosamine residues with free amino groups in cell wall peptidoglycan from bacilli as a factor responsible for resistance to lysozyme. J Bacteriol. 1973 Feb;113(2):592–8.

258. Li S, Wang H, Ma J, Gu G, Chen Z, Guo Z. One-pot four-enzyme synthesis of thymidinediphosphate-l-rhamnose. Chem Commun (Camb). 2016 Nov 29;52(97):13995–8.

259. Steiner T, Lamerz A-C, Hess P, Breithaupt C, Krapp S, Bourenkov G, et al. Open and closed structures of the UDP-glucose pyrophosphorylase from Leishmania major. J Biol Chem. 2007 Apr 27;282(17):13003–10.

260. Breazeale SD, Ribeiro AA, Raetz CRH. Origin of lipid A species modified with 4-amino-4-deoxy-L-arabinose in polymyxin-resistant mutants of Escherichia coli. An aminotransferase (ArnB) that generates UDP-4-deoxyl-L-arabinose. J Biol Chem. 2003 Jul 4;278(27):24731–9.

261. McCarthy TR, Torrelles JB, MacFarlane AS, Katawczik M, Kutzbach B, Desjardin LE, et al. Overexpression of Mycobacterium tuberculosis manB, a phosphomannomutase that increases phosphatidylinositol mannoside biosynthesis in Mycobacterium smegmatis and mycobacterial association with human macrophages. Mol Microbiol. 2005 Nov;58(3):774–90.

262. Roman E, Roberts I, Lidholt K, Kusche-Gullberg M. Overexpression of UDP-glucose dehydrogenase in Escherichia coli results in decreased biosynthesis of K5 polysaccharide. Biochem J. 2003 Sep 15;374(Pt 3):767–72.

263. Breazeale SD, Ribeiro AA, Raetz CRH. Oxidative decarboxylation of UDP-glucuronic acid in extracts of polymyxin-resistant Escherichia coli. Origin of lipid a species modified with. J Biol Chem. 2002 Jan 25;277(4):2886–96.

264. Tonetti M, Zanardi D, Gurnon JR, Fruscione F, Armirotti A, Damonte G, et al. Paramecium bursaria Chlorella virus 1 encodes two enzymes involved in the biosynthesis of GDP-L-fucose and GDP-D-rhamnose. J Biol Chem. 2003 Jun 13;278(24):21559–65.

265. Vessal M, Hassid WZ. Partial Purification and Properties of l-Glutamine d-Fructose 6-Phosphate Amidotransferase from Phaseolus aureus. Plant Physiol. 1972 Jun;49(6):977–81.

266. Li Y, Yu H, Cao H, Muthana S, Chen X. Pasteurella multocida CMP-sialic acid synthetase and mutants of Neisseria meningitidis CMP-sialic acid synthetase with improved substrate promiscuity. Appl Microbiol Biotechnol. 2012 Mar;93(6):2411–23.

267. Li Z, Hwang S, Ericson J, Bowler K, Bar-Peled M. Pen and Pal are nucleotide-sugar dehydratases that convert UDP-GlcNAc to. J Biol Chem. 2015 Jan 9;290(2):691–704.

268. Stray-Pedersen A, Backe PH, Sorte HS, Mørkrid L, Chokshi NY, Erichsen HC, et al. PGM3 mutations cause a congenital disorder of glycosylation with severe immunodeficiency and skeletal dysplasia. Am J Hum Genet. 2014 Jul 3;95(1):96–107.

269. Bandini G, Mariño K, Güther MLS, Wernimont AK, Kuettel S, Qiu W, et al. Phosphoglucomutase is absent in Trypanosoma brucei and redundantly substituted by phosphomannomutase and phospho-N-acetylglucosamine mutase. Mol Microbiol. 2012 Aug;85(3):513–34.

270. Nic Lochlainn L, Caffrey P. Phosphomannose isomerase and phosphomannomutase gene disruptions in Streptomyces nodosus: impact on amphotericin biosynthesis and implications for glycosylation engineering. Metab Eng. 2009 Jan;11(1):40–7.

271. Wells TN, Coulin F, Payton MA, Proudfoot AE. Phosphomannose isomerase from Saccharomyces cerevisiae contains two inhibitory metal ion binding sites. Biochemistry. 1993 Feb 9;32(5):1294–301.

272. Mizanur RM, Pohl NLB. Phosphomannose isomerase/GDP-mannose pyrophosphorylase from Pyrococcus furiosus: a thermostable biocatalyst for the synthesis of guanidinediphosphate-activated and mannose-containing sugar nucleotides. Org Biomol Chem. 2009 May 21;7(10):2135–9.

273. Singh B, Lee C-B, Sohng JK. Precursor for biosynthesis of sugar moiety of doxorubicin depends on rhamnose biosynthetic pathway in Streptomyces peucetius ATCC 27952. Appl Microbiol Biotechnol. 2010 Feb;85(5):1565–74.

274. Bruender NA, Holden HM. Probing the catalytic mechanism of a C-3’-methyltransferase involved in the biosynthesis of D-tetronitrose. Protein Sci. 2012

Jun;21(6):876–86.

275. Rosano C, Bisso A, Izzo G, Tonetti M, Sturla L, De Flora A, et al. Probing the catalytic mechanism of GDP-4-keto-6-deoxy-d-mannose Epimerase/Reductase by kinetic and crystallographic characterization of site-specific mutants. J Mol Biol. 2000 Oct 13;303(1):77–91.

276. Silva E, Marques AR, Fialho AM, Granja AT, Sá-Correia I. Proteins encoded by Sphingomonas elodea ATCC 31461 rmlA and ugpG genes, involved in gellan gum biosynthesis, exhibit both dTDP- and UDP-glucose pyrophosphorylase activities. Appl Environ Microbiol. 2005 Aug;71(8):4703–12.

277. Goon S, Kelly JF, Logan SM, Ewing CP, Guerry P. Pseudaminic acid, the major modification on Campylobacter flagellin, is synthesized via the Cj1293 gene. Mol Microbiol. 2003 Oct;50(2):659–71.

278. Deretic V, Gill JF, Chakrabarty AM. Pseudomonas aeruginosa infection in cystic fibrosis: nucleotide sequence and transcriptional regulation of the algD gene. Nucleic Acids Res. 1987 Jun 11;15(11):4567–81.

279. Patin D, Bayliss M, Mengin-Lecreulx D, Oyston P, Blanot D. Purification and biochemical characterisation of GlmU from Yersinia pestis. Arch Microbiol. 2015 Apr;197(3):371–8.

280. Huynh QK, Gulve EA, Dian T. Purification and characterization of glutamine:fructose 6-phosphate amidotransferase from rat liver. Arch Biochem Biophys. 2000 Jul 15;379(2):307–13.

281. Roychoudhury S, May TB, Gill JF, Singh SK, Feingold DS, Chakrabarty AM. Purification and characterization of guanosine diphospho-D-mannose dehydrogenase. A key enzyme in the biosynthesis of alginate by Pseudomonas aeruginosa. J Biol Chem. 1989 Jun 5;264(16):9380–5.

282. Shinabarger D, Berry A, May TB, Rothmel R, Fialho A, Chakrabarty AM. Purification and characterization of phosphomannose isomerase-guanosine diphospho-D-mannose pyrophosphorylase. A bifunctional enzyme in the alginate biosynthetic pathway of Pseudomonas aeruginosa. J Biol Chem. 1991 Feb 5;266(4):2080–8.

283. Vann WF, Tavarez JJ, Crowley J, Vimr E, Silver RP. Purification and characterization of the Escherichia coli K1 neuB gene product. Glycobiology. 1997 Jul;7(5):697–701.

284. Fernandez-Sorensen A, Carlson DM. Purification and properties of phosphoacetylglucosamine mutase. J Biol Chem. 1971 Jun 10;246(11):3485–93.

285. Ding L, Seto BL, Ahmed SA, Coleman WGJ. Purification and properties of the Escherichia coli K-12 NAD-dependent nucleotide diphosphosugar epimerase,

ADP-L-glycero-D-mannoheptose 6-epimerase. J Biol Chem. 1994 Sep 30;269(39):24384–90.

286. Yamamoto K, Moriguchi M, Kawai H, Tochikura T. Purification and some properties of uridine diphosphate N-acetylglucosamine pyrophosphorylase from Neurospora crassa. Can J Microbiol. 1979 Dec;25(12):1381–6.

287. Lindquist L, Kaiser R, Reeves PR, Lindberg AA. Purification, characterization and HPLC assay of Salmonella glucose-1-phosphate thymidylyl-transferase from the cloned rfbA gene. Eur J Biochem. 1993 Feb 1;211(3):763–70.

288. Tullius MV, Munson RSJ, Wang J, Gibson BW. Purification, cloning, and expression of a cytidine 5’-monophosphate. J Biol Chem. 1996 Jun 28;271(26):15373–80.

289. Vann WF, Silver RP, Abeijon C, Chang K, Aaronson W, Sutton A, et al. Purification, properties, and genetic location of Escherichia coli cytidine 5’-monophosphate N-acetylneuraminic acid synthetase. J Biol Chem. 1987 Dec 25;262(36):17556–62.

290. Jolly L, Ferrari P, Blanot D, Van Heijenoort J, Fassy F, Mengin-Lecreulx D. Reaction mechanism of phosphoglucosamine mutase from Escherichia coli. Eur J Biochem. 1999 May;262(1):202–10.

291. Rhomberg S, Fuchsluger C, Rendić D, Paschinger K, Jantsch V, Kosma P, et al. Reconstitution in vitro of the GDP-fucose biosynthetic pathways of Caenorhabditis elegans and Drosophila melanogaster. FEBS J. 2006 May;273(10):2244–56.

292. Martínez LI, Piattoni CV, Garay SA, Rodrígues DE, Guerrero SA, Iglesias AA. Redox regulation of UDP-glucose pyrophosphorylase from Entamoeba histolytica. Biochimie. 2011 Feb;93(2):260–8.

293. Li P, Liu Q, Huang C, Zhao X, Roland KL, Kong Q. Reversible synthesis of colanic acid and O-antigen polysaccharides in Salmonella Typhimurium enhances induction of cross-immune responses and provides protection against heterologous Salmonella challenge. Vaccine. 2017 May 15;35(21):2862–9.

294. Li W, Xin Y, McNeil MR, Ma Y. rmlB and rmlC genes are essential for growth of mycobacteria. Biochem Biophys Res Commun. 2006 Mar 31;342(1):170–8.

295. Dong C, Major LL, Srikannathasan V, Errey JC, Giraud M-F, Lam JS, et al. RmlC, a C3’ and C5’ carbohydrate epimerase, appears to operate via an intermediate with an unusual twist boat conformation. J Mol Biol. 2007 Jan 5;365(1):146–59.

296. Giraud MF, Leonard GA, Field RA, Berlind C, Naismith JH. RmlC, the third enzyme of dTDP-L-rhamnose pathway, is a new class of epimerase. Nat Struct

Biol. 2000 May;7(5):398–402.

297. Canals R, Jiménez N, Vilches S, Regué M, Merino S, Tomás JM. Role of Gne and GalE in the virulence of Aeromonas hydrophila serotype O34. J Bacteriol. 2007 Jan;189(2):540–50.

298. Liu XD, Duan J, Guo LH. Role of phosphoglucosamine mutase on virulence properties of Streptococcus mutans. Oral Microbiol Immunol. 2009 Aug;24(4):272–7.

299. Pardeshi P, Rao KK, Balaji PV. Rv3634c from Mycobacterium tuberculosis H37Rv encodes an enzyme with UDP-Gal/Glc and. PLoS One. 2017;12(4):e0175193.

300. Hashimoto H, Sakakibara A, Yamasaki M, Yoda K. Saccharomyces cerevisiae VIG9 encodes GDP-mannose pyrophosphorylase, which is essential for protein glycosylation. J Biol Chem. 1997 Jun 27;272(26):16308–14.

301. Effertz K, Hinderlich S, Reutter W. Selective loss of either the epimerase or kinase activity of UDP-N-acetylglucosamine. J Biol Chem. 1999 Oct 1;274(40):28771–8.

302. Zaretsky M, Roine E, Eichler J. Sialic Acid-Like Sugars in Archaea: Legionaminic Acid Biosynthesis in the Halophile Halorubrum sp. PV6. Front Microbiol. 2018;9:2133.

303. Poulin MB, Shi Y, Protsko C, Dalrymple SA, Sanders DAR, Pinto BM, et al. Specificity of a UDP-GalNAc pyranose-furanose mutase: a potential therapeutic target for Campylobacter jejuni infections. Chembiochem. 2014 Jan 3;15(1):47–56.

304. Kiser KB, Bhasin N, Deng L, Lee JC. Staphylococcus aureus cap5P encodes a UDP-N-acetylglucosamine 2-epimerase with functional redundancy. J Bacteriol. 1999 Aug;181(16):4818–24.

305. van der Beek SL, Zorzoli A, Çanak E, Chapman RN, Lucas K, Meyer BH, et al. Streptococcal dTDP-L-rhamnose biosynthesis enzymes: functional characterization and lead compound identification. Mol Microbiol. 2019 Apr;111(4):951–64.

306. Song WS, Nam MS, Namgung B, Yoon S. Structural analysis of PseH, the Campylobacter jejuni N-acetyltransferase involved in bacterial O-linked glycosylation. Biochem Biophys Res Commun. 2015 Mar 20;458(4):843–8.

307. Thoden JB, Schäffer C, Messner P, Holden HM. Structural analysis of QdtB, an aminotransferase required for the biosynthesis of dTDP-3-acetamido-3,6-dideoxy-alpha-D-glucose. Biochemistry. 2009 Feb 24;48(7):1553–61.

308. Chantigian DP, Thoden JB, Holden HM. Structural and biochemical

characterization of a bifunctional ketoisomerase/N-acetyltransferase from Shewanella denitrificans. Biochemistry. 2013 Nov 19;52(46):8374–85.

309. Dorfmueller HC, Fang W, Rao FV, Blair DE, Attrill H, van Aalten DMF. Structural and biochemical characterization of a trapped coenzyme A adduct of Caenorhabditis elegans glucosamine-6-phosphate N-acetyltransferase 1. Acta Crystallogr D Biol Crystallogr. 2012 Aug;68(Pt 8):1019–29.

310. Riegert AS, Thoden JB, Schoenhofen IC, Watson DC, Young NM, Tipton PA, et al. Structural and Biochemical Investigation of PglF from Campylobacter jejuni Reveals a New Mechanism for a Member of the Short Chain Dehydrogenase/Reductase Superfamily. Biochemistry. 2017 Nov 14;56(45):6030–40.

311. Rangarajan ES, Proteau A, Cui Q, Logan SM, Potetinova Z, Whitfield D, et al. Structural and functional analysis of Campylobacter jejuni PseG: a udp-sugar hydrolase from the pseudaminic acid biosynthetic pathway. J Biol Chem. 2009 Jul 31;284(31):20989–1000.

312. Schoenhofen IC, Lunin VV, Julien J-P, Li Y, Ajamian E, Matte A, et al. Structural and functional characterization of PseC, an aminotransferase involved in the biosynthesis of pseudaminic acid, an essential flagellar modification in Helicobacter pylori. J Biol Chem. 2006 Mar 31;281(13):8907–16.

313. Thoden JB, Cook PD, Schäffer C, Messner P, Holden HM. Structural and functional studies of QdtC: an N-acetyltransferase required for the biosynthesis of dTDP-3-acetamido-3,6-dideoxy-alpha-D-glucose. Biochemistry. 2009 Mar 31;48(12):2699–709.

314. Thoden JB, Holden HM. Structural and functional studies of WlbA: A dehydrogenase involved in the biosynthesis of 2,3-diacetamido-2,3-dideoxy-D-mannuronic acid . Biochemistry. 2010 Sep 14;49(36):7939–48.

315. Kubiak RL, Phillips RK, Zmudka MW, Ahn MR, Maka EM, Pyeatt GL, et al. Structural and functional studies on a 3’-epimerase involved in the biosynthesis of dTDP-6-deoxy-D-allose. Biochemistry. 2012 Nov 20;51(46):9375–83.

316. Somoza JR, Menon S, Schmidt H, Joseph-McCarthy D, Dessen A, Stahl ML, et al. Structural and kinetic analysis of Escherichia coli GDP-mannose 4,6 dehydratase provides insights into the enzyme’s catalytic mechanism and regulation by. Structure. 2000 Feb 15;8(2):123–35.

317. Taylor PL, Sugiman-Marangos S, Zhang K, Valvano MA, Wright GD, Junop MS. Structural and kinetic characterization of the LPS biosynthetic enzyme. Biochemistry. 2010 Feb 9;49(5):1033–41.

318. Gunawan J, Simard D, Gilbert M, Lovering AL, Wakarchuk WW, Tanner ME, et al. Structural and mechanistic analysis of sialic acid synthase NeuB from Neisseria

meningitidis in complex with Mn2+, phosphoenolpyruvate, and N-acetylmannosaminitol. J Biol Chem. 2005 Feb 4;280(5):3555–63.

319. Lee M, Sousa MC. Structural basis for substrate specificity in ArnB. A key enzyme in the polymyxin resistance pathway of Gram-negative bacteria. Biochemistry. 2014 Feb 4;53(4):796–805.

320. Roeben A, Plitzko JM, Körner R, Böttcher UMK, Siegers K, Hayer-Hartl M, et al. Structural basis for subunit assembly in UDP-glucose pyrophosphorylase from Saccharomyces cerevisiae. J Mol Biol. 2006 Dec 8;364(4):551–60.

321. Kim H, Choi J, Kim T, Lokanath NK, Ha SC, Suh SW, et al. Structural basis for the reaction mechanism of UDP-glucose pyrophosphorylase. Mol Cells. 2010 Apr;29(4):397–405.

322. Regni C, Naught L, Tipton PA, Beamer LJ. Structural basis of diverse substrate recognition by the enzyme PMM/PGM from P. aeruginosa. Structure. 2004 Jan;12(1):55–63.

323. Hwang T-S, Hung C-H, Teo C-F, Chen G-T, Chang L-S, Chen S-F, et al. Structural characterization of Escherichia coli sialic acid synthase. Biochem Biophys Res Commun. 2002 Jul 5;295(1):167–73.

324. Pelissier M-C, Lesley SA, Kuhn P, Bourne Y. Structural insights into the catalytic mechanism of bacterial guanosine-diphospho-D-mannose pyrophosphorylase and its regulation by divalent ions. J Biol Chem. 2010 Aug 27;285(35):27468–76.

325. Dow GT, Gilbert M, Thoden JB, Holden HM. Structural investigation on WlaRG from Campylobacter jejuni: A sugar aminotransferase. Protein Sci. 2017 Mar;26(3):586–99.

326. Ishiyama N, Creuzenet C, Miller WL, Demendi M, Anderson EM, Harauz G, et al. Structural studies of FlaA1 from Helicobacter pylori reveal the mechanism for inverting 4,6-dehydratase activity. J Biol Chem. 2006 Aug 25;281(34):24489–95.

327. Dow GT, Thoden JB, Holden HM. Structural studies on KijD1, a sugar C-3’-methyltransferase. Protein Sci. 2016 Dec;25(12):2282–9.

328. Gruszczyk J, Fleurie A, Olivares-Illana V, Béchet E, Zanella-Cleon I, Moréra S, et al. Structure analysis of the Staphylococcus aureus UDP-N-acetyl-mannosamine dehydrogenase Cap5O involved in capsular polysaccharide biosynthesis. J Biol Chem. 2011 May 13;286(19):17112–21.

329. Rangarajan ES, Ruane KM, Sulea T, Watson DC, Proteau A, Leclerc S, et al. Structure and active site residues of PglD, an N-acetyltransferase from the bacillosamine synthetic pathway required for N-glycan synthesis in Campylobacter jejuni. Biochemistry. 2008 Feb 19;47(7):1827–36.

330. McCoy JG, Bitto E, Bingman CA, Wesenberg GE, Bannen RM, Kondrashov DA, et al. Structure and dynamics of UDP-glucose pyrophosphorylase from Arabidopsis thaliana with bound UDP-glucose and UTP. J Mol Biol. 2007 Feb 23;366(3):830–41.

331. Zhang Z, Bulloch EMM, Bunker RD, Baker EN, Squire CJ. Structure and function of GlmU from Mycobacterium tuberculosis. Acta Crystallogr D Biol Crystallogr. 2009 Mar;65(Pt 3):275–83.

332. Taylor PL, Blakely KM, de Leon GP, Walker JR, McArthur F, Evdokimova E, et al. Structure and function of sedoheptulose-7-phosphate isomerase, a critical enzyme for lipopolysaccharide biosynthesis and a target for antibiotic adjuvants. J Biol Chem. 2008 Feb 1;283(5):2835–45.

333. Mosimann SC, Gilbert M, Dombroswki D, To R, Wakarchuk W, Strynadka NC. Structure of a sialic acid-activating synthetase, CMP-acylneuraminate synthetase in the presence and absence of CDP. J Biol Chem. 2001 Mar 16;276(11):8190–6.

334. Thoden JB, Goneau M-F, Gilbert M, Holden HM. Structure of a sugar N-formyltransferase from Campylobacter jejuni. Biochemistry. 2013 Sep 3;52(35):6114–26.

335. Rocha J, Popescu AO, Borges P, Mil-Homens D, Moreira LM, Sá-Correia I, et al. Structure of Burkholderia cepacia UDP-glucose dehydrogenase (UGD) BceC and role of Tyr10 in final hydrolysis of UGD thioester intermediate. J Bacteriol. 2011 Aug;193(15):3978–87.

336. Koropatkin NM, Holden HM. Structure of CDP-D-glucose 4,6-dehydratase from Salmonella typhi complexed with. Acta Crystallogr D Biol Crystallogr. 2005 Apr;61(Pt 4):365–73.

337. Kedzierski L, Malby RL, Smith BJ, Perugini MA, Hodder AN, Ilg T, et al. Structure of Leishmania mexicana phosphomannomutase highlights similarities with human isoforms. J Mol Biol. 2006 Oct 13;363(1):215–27.

338. Mulichak AM, Bonin CP, Reiter W-D, Garavito RM. Structure of the MUR1 GDP-mannose 4,6-dehydratase from Arabidopsis thaliana: implications for ligand binding and specificity. Biochemistry. 2002 Dec 31;41(52):15578–89.

339. Barton WA, Lesniak J, Biggins JB, Jeffrey PD, Jiang J, Rajashankar KR, et al. Structure, mechanism and engineering of a nucleotidylyltransferase as a first step toward glycorandomization. Nat Struct Biol. 2001 Jun;8(6):545–51.

340. Sagurthi SR, Gowda G, Savithri HS, Murthy MRN. Structures of mannose-6-phosphate isomerase from Salmonella typhimurium bound to metal atoms and substrate: implications for catalytic mechanism. Acta Crystallogr D Biol Crystallogr. 2009 Jul;65(Pt 7):724–32.

341. Thorson JS, Lo SF, Ploux O, He X, Liu HW. Studies of the biosynthesis of 3,6-dideoxyhexoses: molecular cloning and characterization of the asc (ascarylose) region from Yersinia pseudotuberculosis serogroup VA. J Bacteriol. 1994 Sep;176(17):5483–93.

342. Zou L, Zheng RB, Lowary TL. Studies on the substrate specificity of a GDP-mannose pyrophosphorylase from Salmonella enterica. Beilstein J Org Chem. 2012;8:1219–26.

343. Friedrich V, Janesch B, Windwarder M, Maresch D, Braun ML, Megson ZA, et al. Tannerella forsythia strains display different cell-surface nonulosonic acids: biosynthetic pathway characterization and first insight into biological implications. Glycobiology. 2017 Apr 1;27(4):342–57.

344. Useglio M, Peirú S, Rodríguez E, Labadie GR, Carney JR, Gramajo H. TDP-L-megosamine biosynthesis pathway elucidation and megalomicin a production in Escherichia coli. Appl Environ Microbiol. 2010 Jun;76(12):3869–77.

345. Soldo B, Scotti C, Karamata D, Lazarevic V. The Bacillus subtilis Gne (GneA, GalE) protein can catalyse UDP-glucose as well as. Gene. 2003 Nov 13;319:65–9.

346. Mølhøj M, Verma R, Reiter W-D. The biosynthesis of D-Galacturonate in plants. functional cloning and characterization of a membrane-anchored UDP-D-Glucuronate 4-epimerase from Arabidopsis. Plant Physiol. 2004 Jul;135(3):1221–30.

347. Hwang S, Li Z, Bar-Peled Y, Aronov A, Ericson J, Bar-Peled M. The biosynthesis of UDP-d-FucNAc-4N-(2)-oxoglutarate (UDP-Yelosamine) in Bacillus cereus ATCC 14579: Pat and Pyl, an aminotransferase and an ATP-dependent Grasp protein that ligates 2-oxoglutarate to UDP-4-amino-sugars. J Biol Chem. 2014 Dec 19;289(51):35620–32.

348. Gu X, Bar-Peled M. The biosynthesis of UDP-galacturonic acid in plants. Functional cloning and characterization of Arabidopsis UDP-D-glucuronic acid 4-epimerase. Plant Physiol. 2004 Dec;136(4):4256–64.

349. Hwang H-Y, Horvitz HR. The Caenorhabditis elegans vulval morphogenesis gene sqv-4 encodes a UDP-glucose dehydrogenase that is temporally and spatially regulated. Proc Natl Acad Sci U S A. 2002 Oct 29;99(22):14224–9.

350. Smith DJ, Cooper M, DeTiani M, Losberger C, Payton MA. The Candida albicans PMM1 gene encoding phosphomannomutase complements a Saccharomyces cerevisiae sec 53-6 mutation. Curr Genet. 1992 Dec;22(6):501–3.

351. Schoenhofen IC, Vinogradov E, Whitfield DM, Brisson J-R, Logan SM. The CMP-legionaminic acid pathway in Campylobacter: biosynthesis involving novel. Glycobiology. 2009 Jul;19(7):715–25.

352. Stevenson G, Lee SJ, Romana LK, Reeves PR. The cps gene cluster of Salmonella strain LT2 includes a second mannose pathway: sequence of two genes and relationship to genes in the rfb gene cluster. Mol Gen Genet. 1991 Jun;227(2):173–80.

353. Yu Q, Zheng X. The crystal structure of human UDP-glucose pyrophosphorylase reveals a latch effect that influences enzymatic activity. Biochem J. 2012 Mar 1;442(2):283–91.

354. Peneff C, Mengin-Lecreulx D, Bourne Y. The crystal structures of Apo and complexed Saccharomyces cerevisiae GNA1 shed light on the catalytic mechanism of an amino-sugar N-acetyltransferase. J Biol Chem. 2001 May 11;276(19):16328–34.

355. WILSON DB, HOGNESS DS. THE ENZYMES OF THE GALACTOSE OPERON IN ESCHERICHIA COLI. I. PURIFICATION AND CHARACTERIZATION OF URIDINE DIPHOSPHOGALACTOSE 4-EPIMERASE. J Biol Chem. 1964 Aug;239:2469–81.

356. Mio T, Yabe T, Arisawa M, Yamada-Okabe H. The eukaryotic UDP-N-acetylglucosamine pyrophosphorylases. Gene cloning, protein expression, and catalytic mechanism. J Biol Chem. 1998 Jun 5;273(23):14392–7.

357. Jolly L, Wu S, van Heijenoort J, de Lencastre H, Mengin-Lecreulx D, Tomasz A. The femR315 gene from Staphylococcus aureus, the interruption of which results in reduced methicillin resistance, encodes a phosphoglucosamine mutase. J Bacteriol. 1997 Sep;179(17):5321–5.

358. Campbell RE, Mosimann SC, van De Rijn I, Tanner ME, Strynadka NC. The first structure of UDP-glucose dehydrogenase reveals the catalytic residues necessary for the two-fold oxidation. Biochemistry. 2000 Jun 13;39(23):7012–23.

359. Bonin CP, Freshour G, Hahn MG, Vanzin GF, Reiter W-D. The GMD1 and GMD2 genes of Arabidopsis encode isoforms of GDP-D-mannose 4,6-dehydratase with cell type-specific expression patterns. Plant Physiol. 2003 Jun;132(2):883–92.

360. De Reuse H, Labigne A, Mengin-Lecreulx D. The Helicobacter pylori ureC gene codes for a phosphoglucosamine mutase. J Bacteriol. 1997 Jun;179(11):3488–93.

361. Thoden JB, Holden HM. The molecular architecture of glucose-1-phosphate uridylyltransferase. Protein Sci. 2007 Mar;16(3):432–40.

362. Thoden JB, Holden HM. The molecular architecture of QdtA, a sugar 3,4-ketoisomerase from Thermoanaerobacterium thermosaccharolyticum. Protein Sci. 2014 Jun;23(6):683–92.

363. Vann WF, Daines DA, Murkin AS, Tanner ME, Chaffin DO, Rubens CE, et al.

The NeuC protein of Escherichia coli K1 is a UDP N-acetylglucosamine 2-epimerase. J Bacteriol. 2004 Feb;186(3):706–12.

364. Mergaert P, Van Montagu M, Holsters M. The nodulation gene nolK of Azorhizobium caulinodans is involved in the formation of. FEBS Lett. 1997 Jun 9;409(2):312–6.

365. Piacente F, De Castro C, Jeudy S, Gaglianone M, Laugieri ME, Notaro A, et al. The rare sugar N-acetylated viosamine is a major component of Mimivirus fibers. J Biol Chem. 2017 May 5;292(18):7385–94.

366. Coleman WGJ. The rfaD gene codes for ADP-L-glycero-D-mannoheptose-6-epimerase. An enzyme required for lipopolysaccharide core biosynthesis. J Biol Chem. 1983 Feb 10;258(3):1985–90.

367. Schmidt M, Arnold W, Niemann A, Kleickmann A, Pühler A. The Rhizobium meliloti pmi gene encodes a new type of phosphomannose isomerase. Gene. 1992 Dec 1;122(1):35–43.

368. Hwang H-Y, Horvitz HR. The SQV-1 UDP-glucuronic acid decarboxylase and the SQV-7 nucleotide-sugar transporter may act in the Golgi apparatus to affect Caenorhabditis elegans vulval morphogenesis and embryonic development. Proc Natl Acad Sci U S A. 2002 Oct 29;99(22):14218–23.

369. Beyer S, Mayer G, Piepersberg W. The StrQ protein encoded in the gene cluster for 5’-hydroxystreptomycin of Streptomyces glaucescens GLA.0 is a alpha-D-glucose-1-phosphate cytidylyltransferase (CDP-D-glucose synthase). Eur J Biochem. 1998 Dec 15;258(3):1059–67.

370. King JD, Poon KKH, Webb NA, Anderson EM, McNally DJ, Brisson J-R, et al. The structural basis for catalytic function of GMD and RMD, two closely related enzymes from the GDP-D-rhamnose biosynthesis pathway. FEBS J. 2009 May;276(10):2686–700.

371. Stokes MJ, Güther MLS, Turnock DC, Prescott AR, Martin KL, Alphey MS, et al. The synthesis of UDP-N-acetylglucosamine is essential for bloodstream form trypanosoma brucei in vitro and in vivo and UDP-N-acetylglucosamine starvation reveals a hierarchy in parasite protein glycosylation. J Biol Chem. 2008 Jun 6;283(23):16147–61.

372. Cleasby A, Wonacott A, Skarzynski T, Hubbard RE, Davies GJ, Proudfoot AE, et al. The x-ray crystal structure of phosphomannose isomerase from Candida albicans at 1.7 angstrom resolution. Nat Struct Biol. 1996 May;3(5):470–9.

373. Davis ML, Thoden JB, Holden HM. The x-ray structure of dTDP-4-keto-6-deoxy-D-glucose-3,4-ketoisomerase. J Biol Chem. 2007 Jun 29;282(26):19227–36.

374. Kepes F, Schekman R. The yeast SEC53 gene encodes phosphomannomutase. J

Biol Chem. 1988 Jul 5;263(19):9155–61.

375. Kneidinger B, O'Riordan K, Li J, Brisson J-R, Lee JC, Lam JS. Three highly conserved proteins catalyze the conversion of. J Biol Chem. 2003 Feb 7;278(6):3615–27.

376. Zimmer AL, Thoden JB, Holden HM. Three-dimensional structure of a sugar N-formyltransferase from Francisella tularensis. Protein Sci. 2014 Mar;23(3):273–83.

377. Nakano Y, Suzuki N, Yoshida Y, Nezu T, Yamashita Y, Koga T. Thymidine diphosphate-6-deoxy-L-lyxo-4-hexulose reductase synthesizing dTDP-6-deoxy-L-talose from Actinobacillus actinomycetemcomitans. J Biol Chem. 2000 Mar 10;275(10):6806–12.

378. Creuzenet C, Lam JS. Topological and functional characterization of WbpM, an inner membrane UDP-GlcNAc C6 dehydratase essential for lipopolysaccharide biosynthesis in Pseudomonas aeruginosa. Mol Microbiol. 2001 Sep;41(6):1295–310.

379. Allard STM, Beis K, Giraud MF, Hegeman AD, Gross JW, Wilmouth RC, et al. Toward a structural understanding of the dehydratase mechanism. Structure. 2002 Jan;10(1):81–92.

380. Cook PD, Kubiak RL, Toomey DP, Holden HM. Two site-directed mutations are required for the conversion of a sugar dehydratase into an aminotransferase. Biochemistry. 2009 Jun 16;48(23):5246–53.

381. Li O, Qian C-D, Zheng D-Q, Wang P-M, Liu Y, Jiang X-H, et al. Two UDP-glucuronic acid decarboxylases involved in the biosynthesis of a bacterial exopolysaccharide in Paenibacillus elgii. Appl Microbiol Biotechnol. 2015 Apr;99(7):3127–39.

382. Köplin R, Brisson JR, Whitfield C. UDP-galactofuranose precursor required for formation of the lipopolysaccharide O antigen of Klebsiella pneumoniae serotype O1 is synthesized by the product of the rfbDKPO1 gene. J Biol Chem. 1997 Feb 14;272(7):4121–8.

383. Daenzer JMI, Sanders RD, Hang D, Fridovich-Keil JL. UDP-galactose 4'-epimerase activities toward UDP-Gal and UDP-GalNAc play different roles in the development of Drosophila melanogaster. PLoS Genet. 2012;8(5):e1002721.

384. Keppler OT, Hinderlich S, Langner J, Schwartz-Albiez R, Reutter W, Pawlita M. UDP-GlcNAc 2-epimerase: a regulator of cell surface sialylation. Science. 1999 May 21;284(5418):1372–6.

385. Rösti J, Barton CJ, Albrecht S, Dupree P, Pauly M, Findlay K, et al. UDP-glucose 4-epimerase isoforms UGE2 and UGE4 cooperate in providing UDP-galactose for

cell wall biosynthesis and growth of Arabidopsis thaliana. Plant Cell. 2007 May;19(5):1565–79.

386. Sen M, Shah B, Rakshit S, Singh V, Padmanabhan B, Ponnusamy M, et al. UDP-glucose 4, 6-dehydratase activity plays an important role in maintaining cell wall integrity and virulence of Candida albicans. PLoS Pathog. 2011 Nov;7(11):e1002384.

387. Nakano K, Omura Y, Tagaya M, Fukui T. UDP-glucose pyrophosphorylase from potato tuber: purification and characterization. J Biochem. 1989 Sep;106(3):528–32.

388. Coyne MJ, Fletcher CM, Reinap B, Comstock LE. UDP-glucuronic acid decarboxylases of Bacteroides fragilis and their prevalence in bacteria. J Bacteriol. 2011 Oct;193(19):5252–9.

389. Bulik DA, van Ophem P, Manning JM, Shen Z, Newburg DS, Jarroll EL. UDP-N-acetylglucosamine pyrophosphorylase, a key enzyme in encysting Giardia, is allosterically regulated. J Biol Chem. 2000 May 12;275(19):14722–8.

390. Majumdar S, Ghatak J, Mukherji S, Bhattacharjee H, Bhaduri A. UDPgalactose 4-epimerase from Saccharomyces cerevisiae. A bifunctional enzyme with aldose 1-epimerase activity. Eur J Biochem. 2004 Feb;271(4):753–9.

391. Bosco MB, Machtey M, Iglesias AA, Aleanzi M. UDPglucose pyrophosphorylase from Xanthomonas spp. Characterization of the enzyme kinetics, structure and inactivation related to oligomeric dissociation. Biochimie. 2009 Feb;91(2):204–13.

392. Telser A, Sussman M. Uridine diphosphate galactose-4-epimerase, a developmentally regulated enzyme in the cellular slime mold Dictyostelium discoideum. J Biol Chem. 1971 Apr 10;246(7):2252–7.

393. Blankenfeldt W, Kerr ID, Giraud M-F, McMiken HJ, Leonard G, Whitfield C, et al. Variation on a theme of SDR. dTDP-6-deoxy-L- lyxo-4-hexulose reductase (RmlD) shows a new Mg2+-dependent dimerization mode. Structure. 2002 Jun;10(6):773–86.

394. Zhang H, Zhou Y, Bao H, Liu H. Vi antigen biosynthesis in Salmonella typhi: characterization of. Biochemistry. 2006 Jul 4;45(26):8163–73.

395. Zhao X, Creuzenet C, Bélanger M, Egbosimba E, Li J, Lam JS. WbpO, a UDP-N-acetyl-D-galactosamine dehydrogenase from Pseudomonas aeruginosa serotype O6. J Biol Chem. 2000 Oct 27;275(43):33252–9.

396. Dummitt B, Micka WS, Chang Y-H. Yeast glutamine-fructose-6-phosphate aminotransferase (Gfa1) requires methionine aminopeptidase activity for proper function. J Biol Chem. 2005 Apr 8;280(14):14356–60.
